# Vericiguat and mortality in heart failure and reduced ejection fraction: the VICTOR trial

**DOI:** 10.1093/eurheartj/ehaf655

**Published:** 2025-08-30

**Authors:** Javed Butler, Francesco Fioretti, Ciaran J McMullan, Kevin J Anstrom, Irina Barash, Marc P Bonaca, Maria Borentain, Stefano Corda, Pedro P Teixeira, Justin A Ezekowitz, Davis Gates, Carolyn S P Lam, Eldrin F Lewis, JoAnn Lindenfeld, Robert J Mentz, Christopher M O’Connor, Piotr Ponikowski, Yogesh N V Reddy, Giuseppe M C Rosano, Clara Saldarriaga, Michele Senni, James Udelson, Alessia Urbinati, Vanja Vlajnic, Adriaan A Voors, Aiwen Xing, Mahesh J Patel, Faiez Zannad

**Affiliations:** Department of Medicine, University of Mississippi, 2500 N State St, Jackson, MS 39216, USA; Baylor Scott and White Research Institute, 3434 Live Oak St., Dallas, TX 75204, USA; Baylor Scott and White Research Institute, 3434 Live Oak St., Dallas, TX 75204, USA; Merck & Co., Inc., Rahway, NJ, USA; Department of Biostatistics, University of North Carolina, Chapel Hill, NC, USA; Merck & Co., Inc., Rahway, NJ, USA; Department of Medicine, Colorado Prevention Center Clinical Research, University of Colorado, Aurora, CO, USA; Bayer US LLC, Whippany, NJ, USA; Bayer AG, Basel, Switzerland; Bayer US LLC, Whippany, NJ, USA; Canadian VIGOUR Centre, University of Alberta, Edmonton, AB, Canada; Merck & Co., Inc., Rahway, NJ, USA; National Heart Centre Singapore and Duke-National University, Singapore, Singapore; Department of Medicine, Stanford University School of Medicine, Stanford, CA, USA; Vanderbilt Heart and Vascular Institute, Vanderbilt University Medical Center, Nashville, TN, USA; Duke Clinical Research Institute, Durham, NC, USA; Inova Heart and Vascular Institute, Falls Church, VA, USA; Department of Heart Diseases, Wroclaw Medical University, Wroclaw, Poland; Department of Cardiovascular Medicine, Mayo Clinic, Rochester, MN, USA; San Raffaele Open University of Rome, Rome, Italy; IRCCS San Raffaele Roma, Rome, Italy; Cardio VID Clinic, Medellin, Colombia; Faculty of Medicine, University of Milano-Bicocca, Milan, Italy; Cardiology Division, Cardiovascular Department, Papa Giovanni XXIII Hospital, Bergamo, Italy; Division of Cardiology and the CardioVascular Center, Tufts Medical Center, Boston, MA, USA; Merck & Co., Inc., Rahway, NJ, USA; Bayer US LLC, Whippany, NJ, USA; Department of Cardiology, University of Groningen, University Medical Center Groningen, Groningen, the Netherlands; Merck & Co., Inc., Rahway, NJ, USA; Merck & Co., Inc., Rahway, NJ, USA; CVCT and Inserm, Centre D'Investigations Cliniques-Plurithématique, Universite de Lorraine, CHRU, Nancy, France

**Keywords:** Heart failure with reduced ejection fraction, Vericiguat, Cardiovascular mortality, All-cause mortality, Sudden cardiac death, Heart failure related deaths

## Abstract

**Background and Aims:**

In the VICTOR trial (NCT05093933), vericiguat was neutral for the primary composite endpoint of cardiovascular death or hospitalization for heart failure (HF). VICTOR was powered to independently assess cardiovascular death. This study reports detailed analysis on the effects of vericiguat on mortality.

**Methods:**

VICTOR, a double-blind, placebo-controlled, randomized trial, enrolled 6105 ambulatory patients with HF and reduced ejection fraction (HFrEF) without recent worsening and randomized them to vericiguat or placebo. The main outcome for this analysis was the pre-specified secondary endpoint of cardiovascular death. All-cause death, sudden cardiac death, and death related to HF were also assessed.

**Results:**

Over a median of 19.7 months (inter-quartile range 14.6–25.4), cardiovascular deaths occurred in 292 patients (5.7 deaths per 100 patient-years) and 346 patients (6.8 deaths per 100 patient-years) in the vericiguat and placebo groups, respectively (hazard ratio [HR] 0.83, 95% confidence interval [CI] 0.71–0.97; *P* = .020). Risk of death from any cause was lower with vericiguat vs placebo (377 [7.3 deaths per 100 patient-years] vs 440 [8.6 deaths per 100 patient-years]; HR 0.84, 95% CI 0.74–0.97; *P* = .015). Sudden cardiac death and HF-related deaths were lower with vericiguat vs placebo (1.6 vs 2.2 events per 100 patient-years; HR 0.75, 95% CI 0.56–0.99; *P* = .042 and 1.7 vs 2.4 events per 100 patient-years; HR 0.71, 95% CI 0.54–0.94; *P* = .016, respectively). Lower mortality rates were consistent across subgroups including baseline therapy. Consistent cardiovascular and all-cause mortality benefit was seen across baseline N-terminal pro-B-type natriuretic peptide levels.

**Conclusions:**

In ambulatory well-treated participants with HFrEF, vericiguat was associated with clinically meaningful reductions in the key secondary outcome of cardiovascular death, as well as all-cause mortality.

## Structured Graphical Abstract

**Structured Graphical Abstract:**
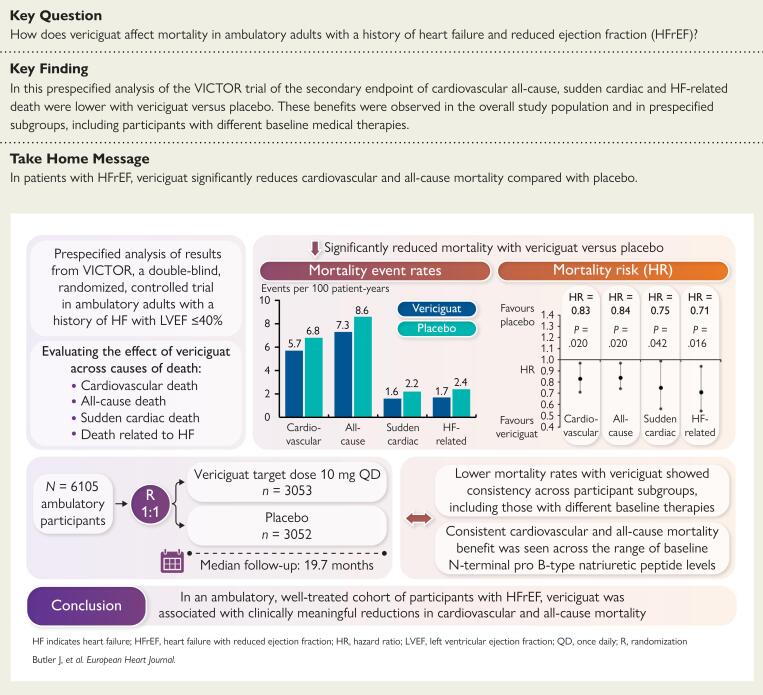
HF indicates heart failure; HFrEF, heart failure with reduced ejection fraction; HR, hazard ratio; LVEF, left ventricular ejection fraction; QD, once daily; R, randomization

**See the editorial comment for this article ‘Vericiguat in heart failure with reduced ejection fraction: being confident in the face of uncertainty', by A. Cannata and M. Packer, https://doi.org/10.1093/eurheartj/ehaf888.**

## Introduction

Despite contemporary treatment with beta-blockers, angiotensin receptor–neprilysin inhibitors (ARNI), mineralocorticoid receptor antagonists (MRA), sodium–glucose cotransporter 2 inhibitors (SGLT2i), and implantable devices, patients with heart failure (HF) with reduced ejection fraction (HFrEF) continue to experience high residual risk for mortality.^1^ Vericiguat is approved for the treatment of worsening HFrEF based on the results of the Vericiguat Global Study in Subjects with Heart Failure with Reduced Ejection Fraction (VICTORIA) trial that showed a significant reduction in the risk for cardiovascular death or hospitalization for HF in patients with HFrEF who were hospitalized for HF within 6 months or needed outpatient intravenous diuretic therapy within 3 months prior to randomization.^2^ Due to the elevated risk profile of patients enrolled in VICTORIA and the high event rate, the overall follow-up was <11 months, diminishing the opportunity to assess the effect of vericiguat on mortality that may require longer follow-up to fully manifest. In a pre-specified analysis of VICTORIA based on N-terminal pro-B-type natriuretic peptide (NT-proBNP) level at baseline, patients in the lower three quartiles (<5314 pg/mL) had a more pronounced overall benefit, including cardiovascular mortality by itself showing a hazard ratio (HR) of 0.78 (95% confidence interval [CI] 0.65–0.94).^3^

In the Vericiguat in Adults with Chronic Heart Failure and Reduced Ejection Fraction (VICTOR) trial, the effect of vericiguat was assessed in ambulatory patients with HFrEF who had not experienced recent worsening, had screening NT-proBNP levels of <6000 pg/mL, and a high percentage of whom were on a background of contemporary guideline-directed medical therapy (GDMT). The primary endpoint of time to cardiovascular death or hospitalization for HF (HR 0.93, 95% CI 0.83–1.04; *P* = .219) or time to first HF hospitalization (HR 0.95, 95% CI 0.82–1.10; *P* = .509) in VICTOR was not significantly reduced.^4^ VICTOR was designed and powered, conditional on the primary endpoint success, to assess the effect of vericiguat on cardiovascular mortality.^5^ The aim of this analysis was to evaluate in detail the effect of vericiguat on mortality outcomes in patients enrolled in VICTOR.

## Methods

### Study design

The VICTOR trial design and participant baseline characteristics have been previously published.^5,6^ Briefly, VICTOR (NCT05093933) was a double-blind, placebo-controlled, 1:1 randomized, event-driven trial assessing the effects of vericiguat (target dose 10 mg daily) vs placebo in ambulatory patients with HFrEF and no recent worsening. The trial was approved by the ethics committee at each site and all patients provided written consent. The key entry criteria included history of HF with left ventricular ejection fraction (LVEF) ≤40%, New York Heart Association (NYHA) class II–IV symptoms on optimally tolerated GDMT and no hospitalization for HF within 6 months or outpatient intravenous diuretic use within 3 months prior to randomization. NT-proBNP levels within 30 days prior to randomization were required to be 600–6000 pg/mL for patients in sinus rhythm and 900–6000 pg/mL for those in atrial fibrillation. Participants with estimated glomerular filtration rate (eGFR) <15 mL/min/1.73 m^2^ were excluded. The study was designed to accrue at least 590 cardiovascular deaths to provide 80% power to detect a HR of 0.80 for this endpoint. Assuming a rate of six cardiovascular deaths per 100 patient-years, a sample size of 6000 was planned to accrue 590 events within 39.5 months from the first patient randomized.

### Outcomes

For this analysis, the main outcome was cardiovascular death, which was the key secondary endpoint in VICTOR. Sudden cardiac death and death related to HF, as components of cardiovascular death, were also assessed individually. Finally, all-cause mortality including both cardiovascular and non-cardiovascular mortality was assessed. All mortality outcomes were independently adjudicated by a blinded clinical events committee.

### Statistical analysis

Baseline characteristics of study participants who experienced cardiovascular death and those who did not were evaluated. Time-to-event endpoints were analysed using the intention-to-treat principle with a stratified log-rank test. Cox-proportional hazards analyses were used to estimate HR with 95% CI with unadjusted nominal *P*-values calculated. However, the proportional hazard assumption was tested using the weighted Schoenfeld residuals test for the time-to-event endpoints. These analyses were not part of a trial hypothesis testing. The impact of vericiguat on sudden cardiac death and death related to HF was assessed individually. The earliest time to death benefit was assessed by performing the stratified log-rank test successively with each accrued mortality event and noting the time when the first unadjusted stratified log-rank *P*-value was <.050. In addition, the effect of vericiguat on cardiovascular mortality in the group that remained on treatment was assessed. An analysis was also performed using the investigator-assigned cause of death. Subgroup analyses were performed to assess consistency of effect across demographics characteristics, comorbidities, eGFR (≥15–≤30 mL/min/1.73 m^2^ vs ≥30–≤60 mL/min/1.73 m^2^ vs ≥60 mL/min/1.73 m^2^), and mean LVEF (<31% vs ≥31%). The impact of baseline medical therapy and implantable cardioverter-defibrillators individually, and the number of GDMT drugs (0–2 vs 3 vs 4) on mortality outcomes were assessed. Outcomes were also assessed based on recency of hospitalization for HF (no prior HF hospitalization, hospitalization for HF 6–12 months, and >12 months prior to randomization). The association with NT-proBNP levels at baseline was assessed as categorical (quartiles) and continuous levels. Continuous variables are summarized as means (standard deviations [SD]) or medians (inter-quartile range) and as counts and percentages for categorical variables. All analyses were conducted using SAS software, version 9.4 (SAS Institute, Cary, NC, USA).

## Results

### Trial participants

Between November 2021 and December 2023, 6105 participants were enrolled, of whom 3053 were assigned to vericiguat and 3052 to placebo. The mean age was 67 years, 23.6% were women, 47.5% of participants had no prior hospitalization for HF, and 14.0% had a history of hospitalization between 6 and 12 months prior to randomization. Overall, 79% of participants had NYHA class II symptoms, and the mean LVEF was 30%. The median NT-proBNP was 1375 pg/mL. Overall, 94.4% participants were on beta-blockers, 93.9% on renin–angiotensin system modulation (including 56.0% on ARNI, 77.7% on MRA, and 59.1% on SGLT2i); 83.4% were treated with at least three medications. Furthermore, 69.8% participants were on loop diuretics at baseline. An implantable cardioverter-defibrillator was present in 32.9% of participants. The median follow-up period (and inter-quartile range) for the primary endpoint was 18.5 (range 13.6–24.7) months and for cardiovascular mortality was 19.7 (range 14.6–25.4) months. Detailed baseline characteristics of the study participants have been published.^6^

### Characteristic of participants by survivorship

Overall, 638 participants experienced cardiovascular death. Baseline characteristics of cardiovascular death survivors and non-survivors are shown in *Table 1*. Non-survivors had a higher proportion of men and those with a history of atrial fibrillation and type 2 diabetes. Median NT-proBNP levels were higher (2194 [range 1198–3683] vs 1314 [range 800–2233] pg/mL) and mean LVEF (29 ± 8% vs 31 ± 7%) and eGFR (66 ± 26 vs 72 ± 24 mL/min/1.73 m^2^) were lower in non-survivors. More non-survivors had an eGFR between 15–30 mL/min/1.73 m^2^ (8% vs 4%) and 30–60 mL/min/1.73 m^2^ (35% vs 30%). Systolic blood pressure (119 ± 16 mmHg vs 121 ± 16 mmHg) was lower in non-survivors. Non-survivors had more advanced symptoms vs survivors (NYHA class III–IV 33% vs 20%), and fewer had no prior history of hospitalization for HF (41% vs 48%). Beta-blockers (92.6% and 94.7%) and MRA (75.5% and 78.0%) were widely used in both non-survivors and survivors, respectively. Loop diuretics were more often prescribed in non-survivors (83.4% vs 68.2%). SGLT2i (50.6% vs 60.1%) and ARNI (50.5% vs 56.6%) use was lower in non-survivors. The proportion of patients with devices was comparable (cardioverter-defibrillators 31.0% and 33.1%; and cardiac resynchronization therapy 15.7% and 14.7%) among non-survivors and survivors, respectively.

**Table 1:** Baseline characteristics of cardiovascular death survivor and non-survivors

Characteristic	Survivors (*N* = 5467)	Non-survivors (*N* = 638)
Age, years, mean (SD)	66.9 (10.9)	67.9 (11.9)
Women, *n* (%)	1307 (23.9)	133 (20.8)
Race, *n* (%)		
White	3526 (64.5)	408 (63.9)
Black/African American^a^	575 (10.5)	81 (12.7)
American Indian or Alaskan	277 (5.1)	24 (3.8)
Native Hawaiian or Pacific Islander	8 (0.1)	3 (0.5)
Asian	663 (12.1)	83 (13.0)
Multi-racial	580 (10.6)	63 (9.9)
Not reported	1 (0.0)	0
Geographic region, *n* (%)		
North America	573 (10.5)	76 (11.9)
Latin and South America	1599 (29.2)	176 (27.6)
Europe		
Eastern Europe	1494 (27.3)	206 (32.3)
Western Europe	1035 (18.9)	92 (14.4)
Asia Pacific	766 (14.0)	88 (13.8)
Comorbidities, *n* (%)		
Diabetes	2288 (41.9)	297 (46.6)
Atrial fibrillation	2045 (37.4)	272 (42.6)
Systolic blood pressure, mmHg, mean (SD)	121.3 (16.1)	118.9 (15.6)
Haemoglobin, g/dL, mean (SD)	14.3 (2.6)	13.8 (1.9)
Body mass index, kg/m^2^, mean (SD)	28.4 (5.6)	28.3 (5.8)
NT-proBNP at randomization, pg/mL, median (IQR)	1314.0 (800.0–2233.0)	2193.5 (1197.5–3683.0)
No prior hospitalization for heart failure, *n* (%)	2635 (48.2)	264 (41.4)
New York Heart Association class, *n* (%)		
II	4393 (80.4)	429 (67.2)
III	1062 (19.4)	206 (32.3)
IV	12 (0.2)	3 (0.5)
Left ventricular ejection fraction, %, mean (SD)	30.6 (6.9)	28.6 (7.7)
eGFR, mL/min/1.73 m^2^, mean (SD)	71.5 (23.8)	65.8 (25.5)
eGFR categories at randomization, *n* (%)		
<15 mL/min/1.73 m^2^	5 (0.1)	2 (0.3)
≥15–<30 mL/min/1.73 m^2^	193 (3.5)	50 (7.8)
≥30 to <60 mL/min/1.73 m^2^	1640 (30.0)	221 (34.6)
≥60 mL/min/1.73 m^2^	3522 (64.4)	353 (55.3)
Medical therapy, *n* (%)		
Loop diuretics	3728 (68.2)	532 (83.4)
Beta-blockers	5175 (94.7)	591 (92.6)
Angiotensin-converting enzyme inhibitor or angiotensin receptor blocker	2070 (37.9)	268 (42.0)
Angiotensin receptor–neprilysin inhibitor	3094 (56.6)	322 (50.5)
Mineralocorticoid receptor antagonist	4266 (78.0)	482 (75.5)
Sodium–glucose cotransporter 2 inhibitor	3287 (60.1)	323 (50.6)
Device therapy, *n* (%)		
Implantable cardioverter-defibrillator	1811 (33.1)	198 (31.0)
Cardiac resynchronization therapy	804 (14.7)	100 (15.7)

eGFR, estimated glomerular filtration rate; IQR, inter-quartile range; NT-proBNP, N-terminal pro-B-type natriuretic peptide; SD, standard deviation.

^a^Includes participants who self-identified as Black, or multi-racial including Black.

### Effect of vericiguat on mortality

**Figure 1:**
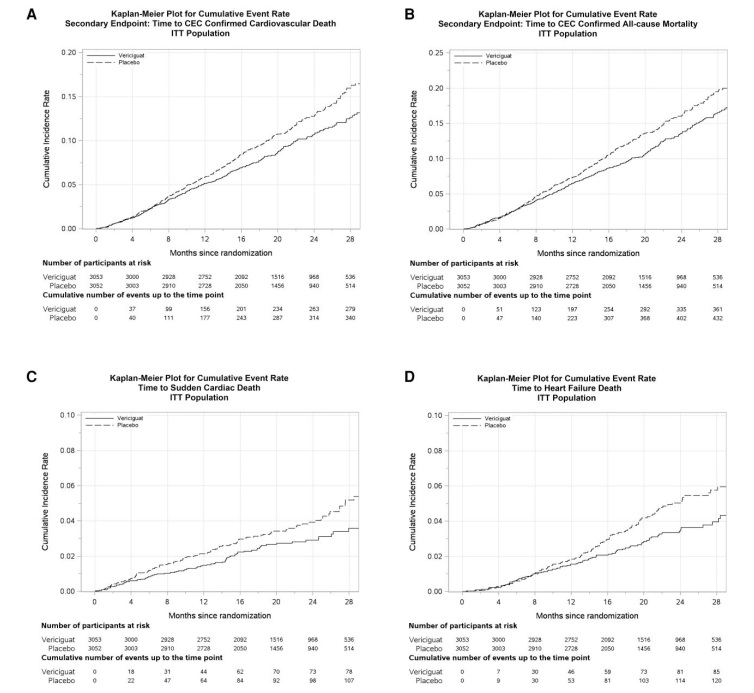
Estimates of the cumulative incidence of the mortality outcomes in the VICTOR trial. Shown are the cumulative incidences of cardiovascular death (*A*), all-cause death (*B*), sudden cardiac death (*C*), and HF-related death (*D*). CI indicates confidence interval; HF, heart failure; HR, hazard ratio

Death from cardiovascular causes occurred in 292 patients (5.7 deaths per 100 patient-years) in the vericiguat group and in 346 (6.8 deaths per 100 patient-years) in the placebo group (HR 0.83, 95% CI 0.71–0.97; *P* = .020). The risk of death from any cause was also lower in the vericiguat group (*N* = 377; 7.3 deaths per 100 patient-years vs *N* = 440 in the placebo group; 8.6 deaths per 100 patient-years; HR 0.84, 95% CI 0.74–0.97; *P* = .015). Both sudden cardiac death and HF-related deaths were lower in the vericiguat group (HR 0.75, 95% CI 0.56–0.99; *P* = .042 and HR 0.71, 95% CI 0.54–0.94; *P* = .016, respectively) (*Table 2* and *Figure 1*). The proportional hazard assumptions passed the weighted Schoenfeld residuals test for the four death outcomes (*P* > .300).

**Table 2:** Mortality outcomes

Variable	Vericiguat (*N* = 3053)		Placebo (*N* = 3052)		Hazard ratio (95% CI)	*P*-value
	No (%) of events	Events/100 patient-years	No (%) of events	Events/100 patient-years		
Cardiovascular death	292 (9.6)	5.7	346 (11.3)	6.8	0.83 (0.71–0.97)	.020
All-cause mortality	377 (12.3)	7.3	440 (14.4)	8.6	0.84 (0.74–0.97)	.015
Sudden cardiac death	83 (2.7)	1.6	110 (3.6)	2.2	0.75 (0.56–0.99)	.042
HF-related death	88 (2.9)	1.7	121 (4.0)	2.4	0.71 (0.54–0.94)	.016

CI, confidence interval; HF, heart failure.

### Time to mortality benefit

The earliest evidence of cardiovascular death benefit, defined when the two-sided *P*-value for cardiovascular death HR decreased below .050, was observed at 11.0 months of median follow-up after an accrual of 335 cardiovascular deaths (150 [4.9%] in the vericiguat group and 185 [6.1%] in the placebo group; HR 0.80, 95% CI 0.65–1.00; *P* = .047). The time to benefit was similar for death from any cause, with the earliest benefit observed at 11.1 months of median follow-up after an accrual of 432 deaths (196 [6.4%] in the vericiguat group and 236 [7.7%] in the placebo group; HR 0.82, 95% CI 0.68–0.995; *P* = .044). The sudden cardiac death benefit was first observed after an accrual of 21 sudden cardiac deaths (6 [0.4%] in the vericiguat group and 15 [1.1%] in the placebo group; HR 0.40, 95% CI 0.155–1.032; *P* = .0497), which occurred at a median follow-up of 5.4 months. The HF death benefit occurred at a median follow-up of 16.8 months after an accrual of 177 HF deaths (76 [2.5%] in the vericiguat group and 101 [3.3%] in the placebo group; HR 0.74, 95% CI 0.55–1.00; *P* = .046).

### Subgroup analysis

**Figure 2:**
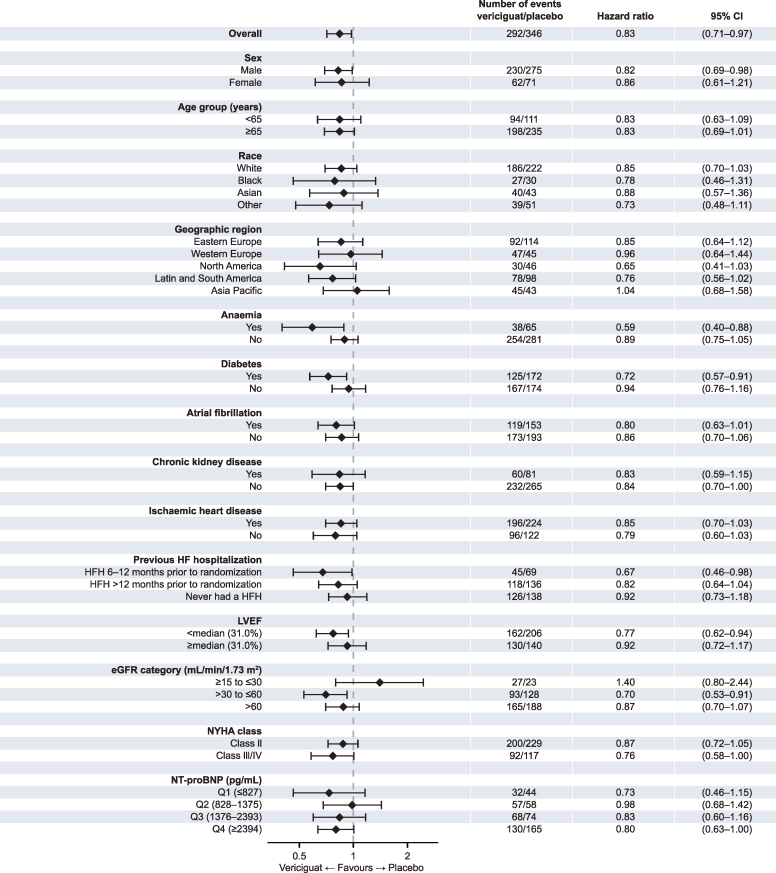
Cardiovascular mortality across subgroups. Shown are the number of events, hazard ratios, and 95% CI across subgroups for cardiovascular death. CI indicates confidence interval; eGFR, estimated glomerular filtration rate; HF, heart failure; HFH, heart failure hospitalization; LVEF, left ventricular ejection fraction; NT-proBNP, N-terminal pro-B-type natriuretic peptide; NYHA, New York Heart Association

**Figure 3:**
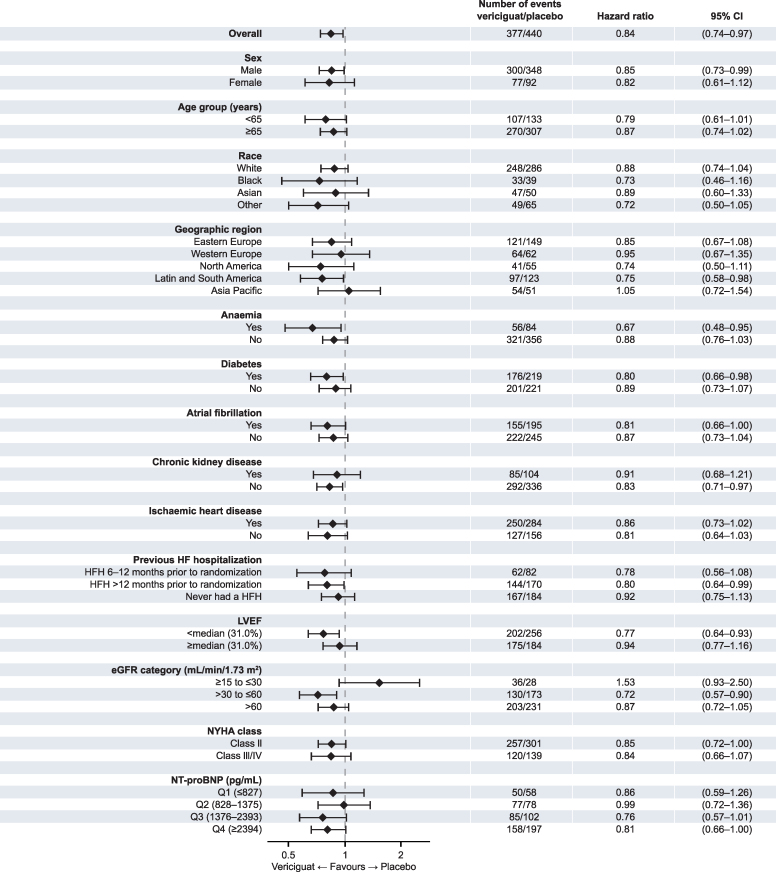
All-cause death across subgroups. Shown are the number of events, hazard ratios, and 95% CI across subgroups for all-cause death. CI indicates confidence interval; eGFR, estimated glomerular filtration rate; HF, heart failure; HFH, heart failure hospitalization; LVEF, left ventricular ejection fraction; NT-proBNP, N-terminal pro-B-type natriuretic peptide; NYHA, New York Heart Association

The benefit of vericiguat on both cardiovascular mortality and all-cause mortality were generally consistent across subgroups based on demographic, region, baseline clinical characteristics, and comorbidities (see Supplementary data online, *Tables S1* and *S2* and *Figures 2* and *3*).

### Prior HF hospitalization

There was no significant difference in benefits on either cardiovascular mortality (interaction *P* = .362) or all-cause mortality (interaction *P* = .562) among patients with no prior history of hospitalization for HF, or prior hospitalization for HF within 6–12 months or more than 12 months prior to randomization (see Supplementary data online, *Tables S1* and *S2* and *Figures 2* and *3*).

### Effect of vericiguat on mortality by baseline therapy

**Figure 4:**
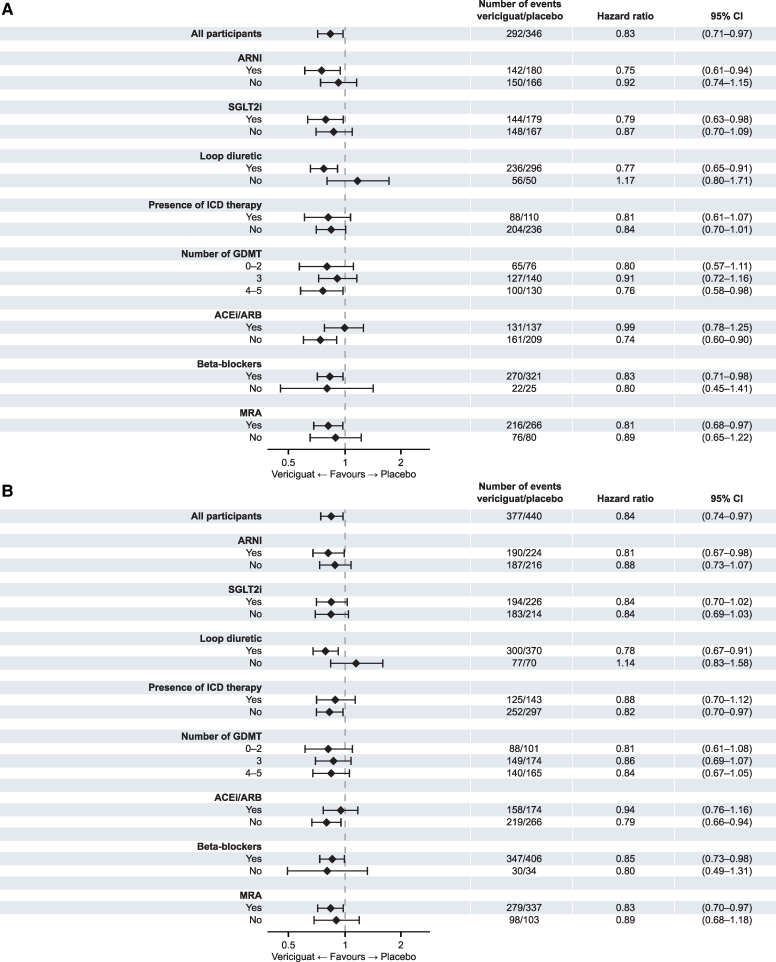
Mortality outcomes based on HF therapy at baseline. Shown are the number of events, hazard ratios, and 95% CIs across subgroups for cardiovascular death (*A*) and all-cause death (*B*). ACEi indicates angiotensin-converting enzyme inhibitor; ARB, angiotensin receptor blocker; CI, confidence interval; GDMT, guideline-directed medical therapy; HF, heart failure; ICD, implantable cardioverter-defibrillator; MRA, mineralocorticoid receptor antagonist; SGLT2i, sodium–glucose cotransporter 2 inhibitor

There was consistent benefit with vericiguat on cardiovascular mortality and all-cause mortality irrespective of baseline therapy with SGLT2i, ARNI, or implantable cardioverter-defibrillator (all treatment-by-outcome interaction *P* > .200). The benefit across the number of GDMT drugs at baseline was consistent for cardiovascular mortality (event rates per 100 patient-years 7.3 vs 9.2 for 0–2 drugs [HR 0.80, 95% CI 0.57–1.11], 6.2 vs 6.8 for 3 drugs [HR 0.91, 95% CI 0.72–1.16], and 4.5 vs 5.9 for 4 drugs [HR 0.76, 95% CI 0.58–0.98] among vericiguat and placebo groups, respectively; treatment-by-outcome interaction *P* = .579). Similar results were seen for all-cause mortality. Event rates were 6.5 vs 8.3 per 100 patient-years and 3.7 vs 3.2 per 100 patient-years among loop-diuretic users and non-users in the vericiguat and placebo groups, respectively (HR 0.77, 95% CI 0.65–0.91 for users and HR 1.17, 95% CI 0.80–1.71 for non-users; treatment-by-outcome interaction *P* = .050). Similar results were seen for all-cause mortality (*Table 3* and *Figure 4*).

**Table 3:** Cardiovascular mortality and all-cause mortality by baseline medical and device therapy

	Vericiguat (*N* = 3053)			Placebo (*N* = 3052)			Treatment comparison	
	*n*/*N* (%)	Events/ 100 patient-years	Events, % (95% CI)	*n*/*N* (%)	Events/ 100 patient-years	Events, % (95% CI)	Hazard ratio (95% CI)	Treatment by subgroup *P*-value
Cardiovascular mortality								
Angiotensin receptor–neprilysin inhibitor								
Yes	142/1734 (8.2)	4.9	12.0 (9.8–14.7)	180/1682 (10.7)	6.4	15.9 (13.4–18.8)	0.75 (0.61–0.94)	.208
No	150/1319 (11.4)	6.7	16.6 (13.9–19.8)	166/1370 (12.1)	7.3	18.1 (15.4–21.3)	0.92 (0.74–1.15)	
Sodium–glucose cotransporter 2 inhibitor								
Yes	144/1812 (7.9)	4.9	11.6 (9.5–14.2)	179/1798 (10.0)	6.1	15.4 (12.9–18.2)	0.79 (0.63–0.98)	.525
No	148/1241 (11.9)	6.8	16.6 (13.9–19.8)	167/1254 (13.3)	7.7	18.6 (15.9–21.8)	0.87 (0.70–1.09)	
Loop diuretic								
Yes	236/2131 (11.1)	6.5	15.5 (13.5–17.9)	296/2129 (13.9)	8.3	20.0 (17.7–22.6)	0.77 (0.65–0.91)	.050
No	56/922 (6.1)	3.7	10.5 (7.3–15.0)	50/923 (5.4)	3.2	9.1 (6.4–12.8)	1.17 (0.80–1.71)	
Implantable cardioverter-defibrillator								
Yes	88/993 (8.9)	5.1	13.0 (10.2–16.4)	110/1016 (10.8)	6.2	15.2 (12.5–18.4)	0.81 (0.61–1.07)	.810
No	204/2060 (9.9)	6.0	14.6 (12.4–17.1)	236/2036 (11.6)	7.1	18.0 (15.5–20.9)	0.84 (0.70–1.01)	
Number of baseline guideline-directed medical therapies								
0–2	65/506 (12.8)	7.3	17.1(13.1–22.1)	76/501 (15.2)	9.2	21.4 (16.8–27.0)	0.80 (0.57–1.11)	.579
3	127/1183 (10.7)	6.2	15.6 (12.9–18.9)	140/1203 (11.6)	6.8	16.4 (13.7–19.6)	0.91 (0.72–1.16)	
4	100/1364 (7.3)	4.5	11.0 (8.6–14.2)	130/1348 (9.6)	5.9	15.6 (12.7–19.1)	0.76 (0.58–0.98)	
Angiotensin-converting enzyme inhibitor/angiotensin receptor blocker								
Yes	131/1150 (11.4)	6.8	16.9 (13.9–20.4)	137/1188 (11.5)	6.9	17.4 (14.5–20.8)	0.99 (0.78–1.25)	.069
No	161/1903 (8.5)	5.0	12.3 (10.2–14.8)	209/1864 (11.2)	6.7	16.5 (14.1–19.2)	0.74 (0.60–0.90)	
Beta-blockers								
Yes	270/2886 (9.4)	5.5	13.8 (12.0–15.8)	321/2880 (11.1)	6.7	16.7 (14.7–18.8)	0.83 (0.71–0.98)	.889
No	22/167 (13.2)	7.7	18.3 (11.4–28.7)	25/172 (14.5)	9.2	20.8 (12.5–33.5)	0.80 (0.45–1.41)	
Mineralocorticoid receptor antagonist								
Yes	216/2358 (9.2)	5.4	13.8 (11.7–16.1)	266/2390 (11.1)	6.7	16.8 (14.7–19.2)	0.81 (0.68–0.97)	.603
No	76/695 (10.9)	6.4	15.0 (11.7–19.2)	80/662 (12.1)	7.2	17.2 (13.5–21.9)	0.89 (0.65–1.22)	
All-cause mortality								
Angiotensin receptor–neprilysin inhibitor								
Yes	190/1734 (11.0)	6.5	16.3 (13.7–19.2)	224/1682 (13.3)	8.0	19.0 (16.4–21.9)	0.81 (0.67–0.98)	.549
No	187/1319 (14.2)	8.4	20.8 (17.8–24.2)	216/1370 (15.8)	9.5	22.6 (19.6–25.9)	0.88 (0.73–1.07)	
Sodium–glucose cotransporter 2 inhibitor								
Yes	194/1812 (10.7)	6.5	16.6 (14.0–19.7)	226/1798 (12.6)	7.7	18.6 (16.0–21.4)	0.84 (0.70–1.02)	1.000
No	183/1241 (14.7)	8.4	20.2 (17.3–23.5)	214/1254 (17.1)	9.9	22.9 (20.0–26.3)	0.84 (0.69–1.03)	
Loop diuretics								
Yes	300/2131 (14.1)	8.3	20.0 (17.7–22.6)	370/2129 (17.4)	10.4	24.0 (21.5–26.6)	0.78 (0.67–0.91)	.037
No	77/922 (8.4)	5.1	13.9 (10.3–18.6)	70/923 (7.6)	4.5	12.2 (9.2–16.1)	1.14 (0.83–1.58)	
Implantable cardioverter-defibrillator								
Yes	125/993 (12.6)	7.2	18.6 (15.3–22.6)	143/1016 (14.1)	8.0	18.9 (16.0–22.3)	0.88 (0.70–1.12)	.639
No	252/2060 (12.2)	7.4	18.0 (15.6–20.6)	297/2036 (14.6)	9.0	21.8 (19.1–24.8)	0.82 (0.70–0.97)	
Number of baseline guideline-directed medical therapies								
0–2	88/506 (17.4)	9.9	22.3 (18.0–27.5)	101/501 (20.2)	12.2	26.5 (21.7–32.1)	0.81 (0.61–1.08)	.950
3	149/1183 (12.6)	7.3	18.1 (15.2–21.5)	174/1203 (14.5)	8.4	20.0 (17.1–23.3)	0.86 (0.69–1.07)	
4	140/1364 (10.3)	6.3	17.0 (13.8–21.0)	165/1348 (12.2)	7.5	18.8 (15.8–22.3)	0.84 (0.67–1.05)	
Angiotensin-converting enzyme inhibitor/angiotensin receptor blocker								
Yes	158/1150 (13.7)	8.2	20.9 (17.6–24.7)	174/1188 (14.6)	8.7	21.5 (18.4–25.1)	0.94 (0.76–1.16)	.224
No	219/1903 (11.5)	6.8	16.7 (14.3–19.4)	266/1864 (14.3)	8.6	19.9 (17.5–22.7)	0.79 (0.66–0.94)	
Beta-blocker								
Yes	347/2886 (12.0)	7.1	18.0 (15.9–20.2)	406/2880 (14.1)	8.4	20.2 (18.2–22.4)	0.85 (0.73–0.98)	.841
No	30/167 (18.0)	10.5	23.5 (16.1–33.6)	34/172 (19.8)	12.5	28.3 (19.0–40.8)	0.80 (0.49–1.31)	
Mineralocorticoid receptor antagonist								
Yes	279/2358 (11.8)	7.0	18.1 (15.8–20.7)	337/2390 (14.1)	8.5	20.6 (18.3–23.1)	0.83 (0.71–0.97)	.638
No	98/695 (14.1)	8.3	19.0 (15.4–23.3)	103/662 (15.6)	9.3	20.8 (16.9–25.5)	0.89 (0.68–1.18)	

### Effect of vericiguat on mortality by baseline natriuretic peptide levels

**Figure 5:**
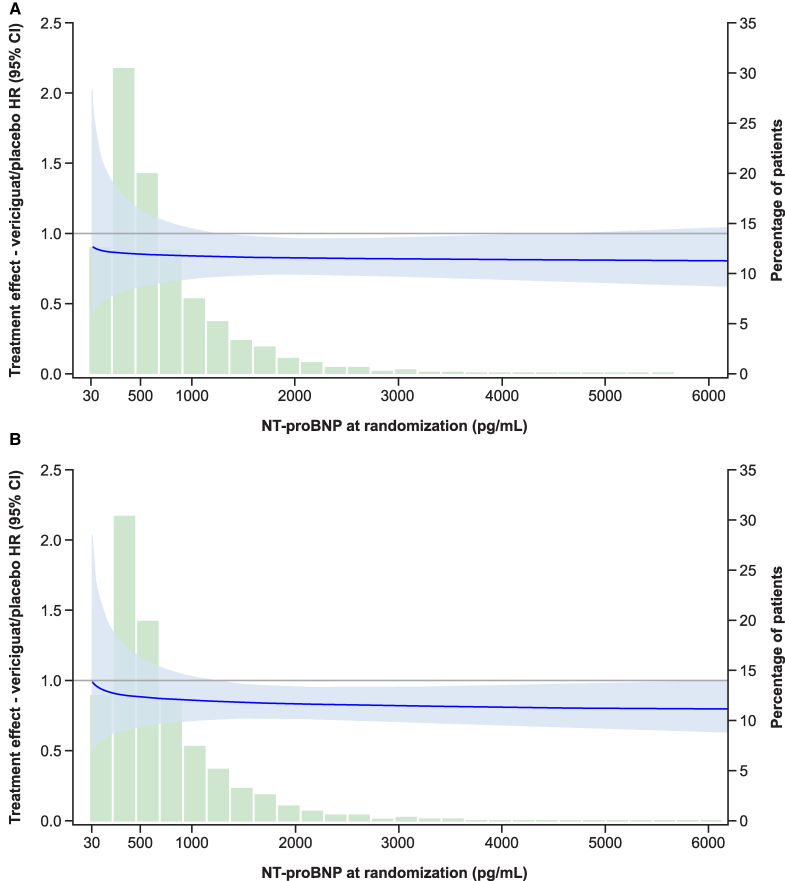
Treatment effect of vericiguat compared with placebo across the range of NT-proBNP. Shown are cardiovascular death (*A*) and all-cause death (*B*). Left axis indicates the treatment effect expressed as HR (curved solid line) with 95% CIs (central shaded area). Vertical shaded bars indicate the number of patients within each NT-proBNP measurement bracket, expressed as a percentage on the right vertical axis. CI indicates confidence interval; HR, hazard ratio; NT-proBNP, N-terminal pro-B-type natriuretic peptide

The beneficial effect of vericiguat on cardiovascular and all-cause mortality was consistent across the quartiles of baseline NT-proBNP concentrations (treatment by quartile interaction *P* ≥ .640). When examined as a continuous variable, vericiguat reduced cardiovascular and all-cause mortality risk across the range of NT-proBNP levels (*Table 2* and *Figure 5*).

### On-treatment analysis

In the on-treatment analysis, a total of 185 cardiovascular deaths occurred in the treatment group vs 239 events in the placebo group (6.1%; 4.0 events per 100 patient-years vs 7.8%; 5.2 events per 100 patient-years; HR 0.77, 95% CI 0.63–0.93; *P* = .007).

### Investigator-assessed cardiovascular death

A total of 284 investigator-reported cardiovascular deaths occurred in the treatment group vs 350 in the placebo group (9.3% vs 11.5%; HR 0.80, 95% CI 0.68–0.93).

## Discussion

In the VICTOR trial, vericiguat led to a 17% relative risk reduction for cardiovascular death and a 16% relative risk reduction for all-cause death (both *P* < .050). Similarly, 25% and 29% relative risk reductions were seen for sudden cardiac death and death due to HF, respectively (both *P* < .050) (*Structured Graphical Abstract*). These data are notable considering that these risk reductions with vericiguat were seen in a cohort of patients receiving the most comprehensive HFrEF evidence-based therapy seen in any HF clinical trial or contemporary real-world registry. Importantly, both the cardiovascular and all-cause death benefits were consistent across major subgroups.

Patients with HFrEF are at substantial risk for mortality despite advances in therapy. In VICTORIA, vericiguat was shown to reduce the risk of cardiovascular death or hospitalization for HF in patients with HFrEF and worsening HF.^2^ Cardiovascular death by itself, however, was not significantly reduced in VICTORIA, where the average follow-up was short, diminishing the chances of seeing a mortality benefit. Longer-term follow-up may generally be needed to assess the impact of HF therapy on mortality. VICTOR was designed and powered to assess the impact of vericiguat on cardiovascular mortality, which was reduced with clinically meaningfully relative risk reductions similar to those seen previously with ARNIs and SGLT2is. Subgroup and sensitivity analyses further confirmed the robustness of the findings. These data underscore the relevance of mortality risk in patients with HFrEF in the contemporary era despite ambulatory status, no recent clinical instability, and on an appropriate baseline medical therapy.

VICTOR stands out with respect to the comprehensive baseline medical therapy in the study population. Despite that, there was consistent benefit with vericiguat on cardiovascular mortality and all-cause mortality irrespective of baseline therapy with SGLT2i, ARNI, MRA, and implantable cardioverter-defibrillators. Importantly, the benefit was consistent regardless of the number of GDMT drugs the participant was receiving at baseline. The lack of treatment heterogeneity observed across subgroups based on baseline therapy in VICTOR reinforces the independent benefit of vericiguat on mortality regardless of other therapies. In this respect, based on tolerability, side-effects, accessibility, or other concerns with current standard of care for patients with ambulatory HFrEF, vericiguat can be an option when indicated without any specific sequencing of drugs necessary. This is further complemented by the consistent safety and tolerability data with vericiguat seen across both VICTOR and VICTORIA.^2,4,7–9^

While the mortality results in VICTOR were positive, the primary composite endpoint of time to cardiovascular death or hospitalization for HF was not statistically significantly reduced. Cardiovascular death is arguably the most important outcome in HF trials and in previous years was the primary outcome of interest. Subsequently the move towards composite endpoints including hospitalization for HF was adopted, in part driven to improve upon the trial logistics, costs, duration, and feasibility. The choice of the primary endpoint is largely arbitrary and in retrospect, we may have chosen cardiovascular mortality as a primary endpoint and the composite of cardiovascular mortality and HF hospitalization as the first key secondary. While the primary endpoint was neutral, it should be noted that VICTOR was powered to assess cardiovascular death effect and the observed cardiovascular death benefit was based on over 600 events. These results were internally consistent, showing improvements in the risk for all-cause mortality as well as specifically in both sudden cardiac death and death due to HF, along with cardiovascular death. The cardiovascular death benefit was also consistent across subgroups. Cardiovascular death is less ambiguous than other endpoints like hospitalization that may be influenced by clinical choices and regional differences.

When a clinical trial is neutral for its primary endpoint, a more nuanced interpretation of secondary endpoint is warranted based on study design, importance of the secondary outcome, statistical power, number of events, magnitude and direction of the treatment effect and CIs, and mechanistic plausibility.^10^ The consistency of result on cardiovascular death, all-cause death, sudden death, and death related to HF, and benefit across subgroups, including those on GDMT in a trial designed and powered to assess the effect of vericiguat on cardiovascular death, provide valuable information for the use of vericiguat in patients with HFrEF.

Participants who died from cardiovascular causes had markers of more advanced disease burden, including higher NT-proBNP level, lower eGFR, more advanced symptoms, higher use of loop diuretics, and higher comorbidity burden. Underutilization of GDMT among non-survivors may have contributed to the worse prognosis; however, patients with more advanced diseases are usually more intolerant to GDMT.^11,12^ The more common use of loop diuretic therapy likely reflects higher baseline clinical and/or subclinical congestion or diuretic resistance, which are predictors of worse outcomes.^13^

The benefit of vericiguat on either cardiovascular mortality or all-cause mortality did not differ significantly among patients with no prior history of hospitalization for HF, or prior hospitalization for HF within 6–12 months or more than 12 months prior to randomization. These results underscore the persistent risk for adverse outcomes in patients with chronic HFrEF irrespective of baseline medical therapy or stability status, and the need for timely interventions to improve outcomes regardless of symptom burden or worsening HF events.

In VICTORIA, the benefit of vericiguat was influenced by baseline NT-proBNP levels.^2,3,14^ Vericiguat reduced the risk of cardiovascular death or hospitalization for HF in patients with NT-proBNP levels up to ∼8000 pg/mL. There was a significant interaction between NT-proBNP levels and treatment effect, with the greatest benefit seen in patients with baseline NT-proBNP levels in the lowest three quartiles (<5314 pg/mL). Based on these data, patients with NT-proBNP levels above 6000 pg/mL at screening were excluded from VICTOR.^5^ Notably, the beneficial effect of vericiguat on cardiovascular and all-cause mortality in VICTOR was consistent regardless of baseline NT-proBNP.

This study is limited by the fact that the primary endpoint of the main trial was neutral and therefore any significant findings in secondary endpoints must be interpreted with caution. The exclusion of patients with NT-proBNP >6000 pg/mL at screening in VICTOR limits the generalizability of these data. Also, the trial was not powered to assess interaction within subgroups. The lack of treatment heterogeneity observed across subgroups based on baseline therapy in VICTOR may suggest an independent benefit of vericiguat on mortality regardless of other therapies. However, these subgroup analyses are exploratory in nature and were not powered to detect interaction effects.

In conclusion, in ambulatory patients on a background of high use of contemporary GDMT, there is a persistent high risk of mortality in patients with HFrEF. In VICTOR, vericiguat was associated with a significant relative risk reduction for cardiovascular death and all-cause death. The risk for both common causes of cardiovascular death in patients with HFrEF, i.e. sudden cardiac deaths and HF-related deaths, were reduced. The observed benefit was consistent across key subgroups. In combination with the known safety and tolerability profile of vericiguat, these findings support the role of vericiguat as an effective addition to GDMT in HFrEF.

## Supplementary Material

ehaf655_Supplementary_Data

## Declarations

### Data Availability

The data sharing policy, including procedures and restrictions, of Merck Sharp & Dohme LLC, a subsidiary of Merck & Co., Inc., Rahway, NJ, USA (MSD), is available at https://externaldatasharing-msd.com/.

### Pre-registered Clinical Trial Number

ClinicalTrials.gov (NCT05093933).
