# Correction to: Vericiguat and mortality in heart failure and reduced ejection fraction: the VICTOR trial

**DOI:** 10.1093/eurheartj/ehag107

**Published:** 2026-04-16

This is a correction to: Javed Butler, Francesco Fioretti, Ciaran J McMullan, Kevin J Anstrom, Irina Barash, Marc P Bonaca, Maria Borentain, Stefano Corda, Pedro P Teixeira, Justin A Ezekowitz, Davis Gates, Carolyn S P Lam, Eldrin F Lewis, JoAnn Lindenfeld, Robert J Mentz, Christopher M O'Connor, Piotr Ponikowski, Yogesh N V Reddy, Giuseppe M C Rosano, Clara Saldarriaga, Michele Senni, James Udelson, Alessia Urbinati, Vanja Vlajnic, Adriaan A Voors, Aiwen Xing, Mahesh J Patel, Faiez Zannad, VICTOR Study Group, Vericiguat and mortality in heart failure and reduced ejection fraction: the VICTOR trial, *European Heart Journal*, Volume 47, Issue 6, 7 February 2026, Pages 683–697, https://doi.org/10.1093/eurheartj/ehaf655

Various corrections were made to Figure 1. Figure 1 should read:

**Figure:**
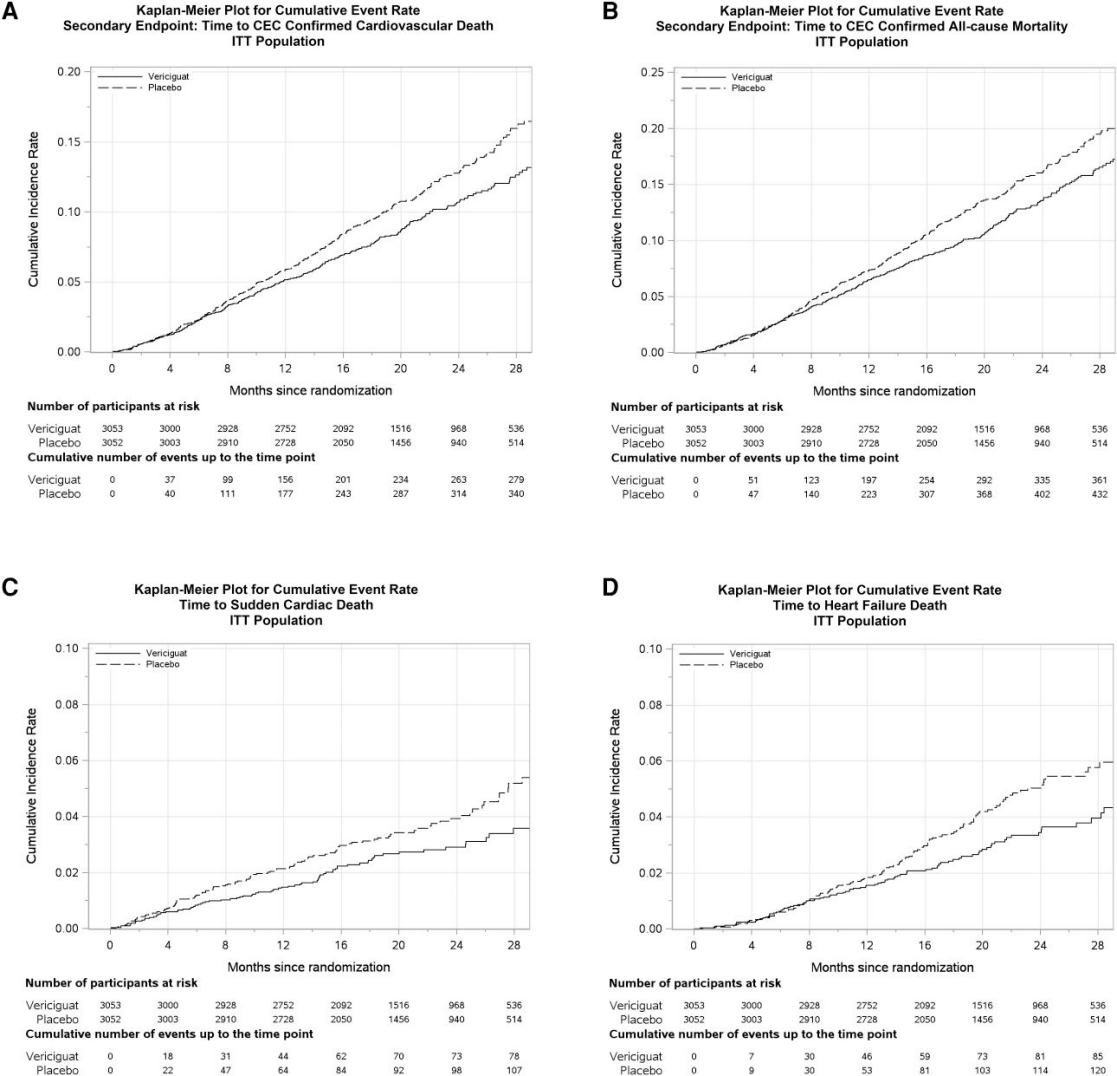


instead of:

**Figure:**
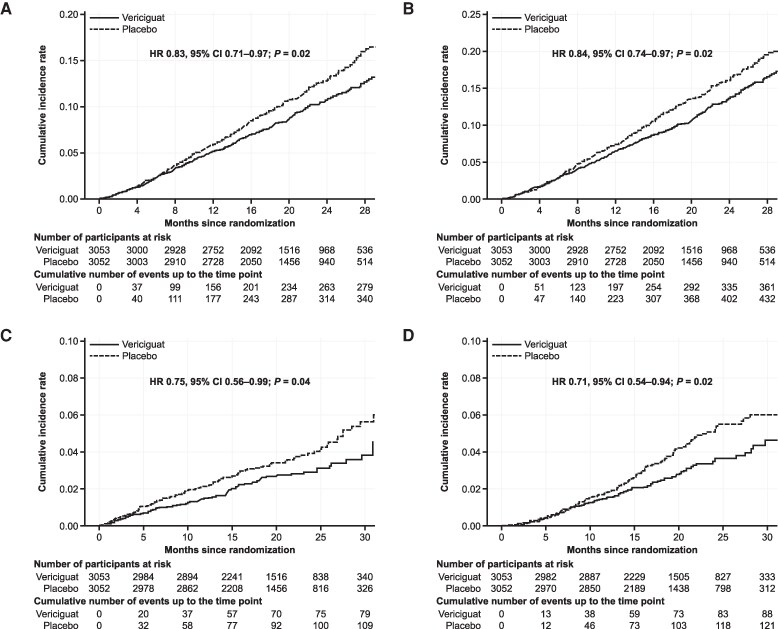


In both Panels C and D, time points on the x-axis were changed from 0, 5, 10, 15, 20, 25, and 30 months to 0, 4, 8, 12, 16, 20, 24, and 28 months. The change in time points and addition of new time point resulted in changes in the numbers of participants at risk and the cumulative number of events up to the time points listed.

In Panels C and D, the numbers of participants at risk in participants randomized to vericiguat were both changed to: 3053, 3000, 2928, 2752, 2092, 1516, 968, and 536.

Original numbers:

3053, 2984, 2894, 2241, 1516, 838, and 340 (Panel C)

3053, 2982, 2887, 2229, 1505, 827, and 333 (Panel D)

In Panels C and D, the numbers of participants at risk in participants randomized to placebo were changed to: 3052, 3003, 2910, 2728, 2050, 1456, 940, and 514

Original numbers:3052, 2978, 2862, 2208, 1456, 816, and 326 (Panel C)

3052, 2970, 2850, 2189, 1438, 798, and 312 (Panel D)

In Panel C, the cumulative number of events up to the time point listed in participants randomized to vericiguat was changed from 0, 20, 37, 57, 70, 75, and 79 to 0, 18, 31, 44, 62, 70, 73, and 78.

In Panel C, the cumulative number of events up to the time point listed in participants randomized to placebo was changed from 0, 32, 58, 77, 92, 100, and 109 to 0, 22, 47, 64, 84, 92, 98, and 107.

In Panel D, the cumulative number of events up to the time point listed in participants randomized to vericiguat was changed from 0, 13, 38, 59, 73, 83, and 88 to 0, 7, 30, 46, 59, 73, 81, and 85.

In Panel D, the cumulative number of events up to the time point listed in participants randomized to placebo was changed from 0, 12, 46, 73, 103, 118, and 121 to 0, 9, 30, 53, 81, 103, 114, and 120.

There were errors in anaemia rows in two tables in the Supplemental material – these have been corrected. A statement was added to the Funding section to read: “GMCR was supported by the Italian Ministry of Health (Ricerca Corrente) 20/1819”.

Affiliations for co-author: “Giuseppe M. C. Rosano^16,17^”

^16^Department of Human Sciences and Promotion of Quality of Life, San Raffaele Open University of Rome, Rome, Italy; ^17^Cardiology, San Raffaele Cassino Hospital, Cassino, Italy; ^18^IRCCS San Raffaele Roma, Rome, Italy;" are corrected to read: “Giuseppe M. C. Rosano^16,17^”, and in the affiliations list: “^16^San Raffaele Open University of Rome, Rome, Italy; ^17^IRCCS San Raffaele Roma, Rome, Italy;”. The affected enumerations of affiliations (originally 19-23) are respectively renumbered within the author and affiliations lists.

