# Supplementary material for: Vericiguat and mortality in heart failure and reduced ejection fraction: the VICTOR trial
Source: Eur Heart J. 2025 Aug 30;47(6):683–97. doi: 10.1093/eurheartj/ehaf655 (PMC13089535; doi:10.1093/eurheartj/ehaf655)
Supplement: ehaf655_Supplementary_Data [file ehaf655_Supplementary_Data.zip › Subgroup_Supplemental_Tables_0919clean.docx]

**Table S1. Mortality Subgroup Analysis: Time-to Cardiovascular Mortality**

|  | **Vericiguat (n=3,053)** | | | **Placebo (n=3,052)** | | | **Treatment Comparison** | |
| --- | --- | --- | --- | --- | --- | --- | --- | --- |
|  | n/m  (%) | Events/  100 PY | KM %  (95% CI) | n/m  (%) | Events/  100 PY | KM %  (95% CI) | HR  (95% CI) | Treatment by Subgroup P-Value |
| **Overall** | | | | | | | | |
|  | 292 /3053  (9.6) | 5.7 | 14.1  (12.3, 16.1) | 346 /3052 (11.3) | 6.8 | 16.9  (15.0, 19.0) | 0.83  (0.71, 0.97) |  |
| **Sex** | | | | | | | | |
| Male | 230/2326  (9.9) | 5.9 | 14.6  (12.6, 17.0) | 275/2339 (11.8) | 7.0 | 17.0  (14.9, 19.3) | 0.82 (0.69,0.98) | 0.808 |
| Female | 62/727  (8.5) | 5.1 | 12.3  (9.1, 16.4) | 71/713 (10.0) | 6.0 | 16.3  (12.4, 21.3) | 0.86 (0.61,1.21) |  |
| **Age Group (Years)** | | | | | | | | |
| < 65 | 94/1109  (8.5) | 5.0 | 12.8  (9.9, 16.5) | 111/1102 (10.1) | 6.0 | 15.4  (12.4, 19.1) | 0.83 (0.63,1.09) | 0.965 |
| ≥ 65 | 198/1944  (10.2) | 6.0 | 14.8  (12.6, 17.3) | 235/1950 (12.1) | 7.2 | 17.7  (15.4, 20.3) | 0.83 (0.69,1.01) |  |
| **Race** | | | | | | | | |
| White | 186/1942  (9.6) | 5.5 | 13.2  (11.2, 15.4) | 222/1992 (11.1) | 6.5 | 16.5  (14.3, 19.0) | 0.85 (0.70,1.03) | 0.913 |
| Black | 27/251  (10.8) | 6.9 | 17.5  (10.9, 27.5) | 30/218 (13.8) | 8.9 | 23.2  (15.6, 33.8) | 0.78 (0.46,1.31) |  |
| Asian | 40/383  (10.4) | 6.6 | 14.3  (10.2, 20.0) | 43/363 (11.8) | 7.4 | 14.6  (10.7, 19.8) | 0.88 (0.57,1.36) |  |
| Other | 39/476  (8.2) | 5.1 | 16.9  (11.0, 25.6) | 51/479 (10.6) | 6.9 | 16.6  (12.0, 22.8) | 0.73 (0.48,1.11) |  |
| **Geographic Region** | | | | | | | | |
| Eastern Europe | 92/823  (11.2) | 6.4 | 14.8  (11.8, 18.6) | 114/877 (13.0) | 7.6 | 19.7  (16.2, 23.7) | 0.85 (0.64,1.12) | 0.557 |
| Western Europe | 47/586  (8.0) | 4.5 | 11.4  (8.2, 15.8) | 45/541  (8.3) | 4.6 | 11.6  (8.4, 15.9) | 0.96 (0.64,1.44) |  |
| North America | 30/324  (9.3) | 5.5 | 14.7  (9.9, 21.7) | 46/325 (14.2) | 8.5 | 20.1  (14.8, 26.9) | 0.65 (0.41,1.03) |  |
| Latin and South America | 78/888  (8.8) | 5.4 | 14.3  (10.8, 18.8) | 98/887 (11.0) | 7.0 | 17.5  (13.7, 22.1) | 0.76 (0.56,1.02) |  |
| Asia Pacific | 45/432  (10.4) | 6.5 | 14.5  (10.5, 19.9) | 43/422 (10.2) | 6.3 | 12.5  (9.1, 16.9) | 1.04 (0.68,1.58) |  |
| **Number of Baseline GDMT** | | | | | | | | |
| 0–2 | 65/506  (12.8) | 7.3 | 17.1  (13.1, 22.1) | 76/501 (15.2) | 9.2 | 21.4  (16.8, 27.0) | 0.80 (0.57,1.11) | 0.579 |
| 3 | 127/1183  (10.7) | 6.2 | 15.6  (12.9, 18.9) | 140/1203 (11.6) | 6.8 | 16.4  (13.7, 19.6) | 0.91 (0.72,1.16) |  |
| 4 | 100/1364  (7.3) | 4.5 | 11.0  (8.6, 14.2) | 130/1348 (9.6) | 5.9 | 15.6  (12.7, 19.1) | 0.76 (0.58,0.98) |  |
| **Anemia** | | | | | | | | |
| Yes | 38/355  (10.7) | 6.3 | 15.5  (10.9, 21.9) | 65/398 (16.3) | 10.4 | 23.9  (18.2, 31.1) | 0.59 (0.40,0.88) | 0.067 |
| No | 254/2698  (9.4) | 5.6 | 13.9  (12.0, 16.0) | 281/2654 (10.6) | 6.3 | 15.9  (14.0, 18.1) | 0.89 (0.75,1.05) |  |
| **Diabetes** | | | | | | | | |
| Yes | 125/1274  (9.8) | 5.8 | 13.5  (11.1, 16.3) | 172/1311 (13.1) | 7.9 | 19.5  (16.5, 23.0) | 0.72 (0.57,0.91) | 0.106 |
| No | 167/1779  (9.4) | 5.6 | 14.5  (12.0, 17.4) | 174/1741 (10.0) | 6.0 | 14.9  (12.6, 17.5) | 0.94 (0.76,1.16) |  |
| **Atrial Fibrillation** | | | | | | | | |
| Yes | 119/1135  (10.5) | 6.1 | 14.6  (12.0, 17.7) | 153/1182 (12.9) | 7.6 | 18.4  (15.6, 21.7) | 0.80 (0.63,1.01) | 0.623 |
| No | 173/1918  (9.0) | 5.4 | 13.8  (11.5, 16.5) | 193/1870 (10.3) | 6.3 | 15.8  (13.4, 18.7) | 0.86 (0.70,1.06) |  |
| **Chronic Kidney Disease** | | | | | | | | |
| Yes | 60/520  (11.5) | 7.0 | 16.3  (12.1, 21.8) | 81/566 (14.3) | 8.6 | 21.4  (17.0, 26.8) | 0.83 (0.59,1.15) | 0.934 |
| No | 232/2533  (9.2) | 5.4 | 13.6  (11.7, 15.8) | 265/2486 (10.7) | 6.4 | 15.8  (13.8, 18.1) | 0.84 (0.70,1.00) |  |
| **Ischemic Heart Disease** | | | | | | | | |
| Yes | 196/1965  (10.0) | 5.8 | 14.2  (12.1, 16.7) | 224/1981 (11.3) | 6.7 | 16.3  (14.1, 18.8) | 0.85 (0.70,1.03) | 0.637 |
| No | 96/1088  (8.8) | 5.4 | 13.8  (10.8, 17.6) | 122/1071 (11.4) | 6.9 | 18.1  (14.7, 22.0) | 0.79 (0.60,1.03) |  |
| **Recency of HFH prior to randomization** | | | | | | | | |
| HFH 6-12 months | 45/424 (10.6) | 6.7 | 15.7 (11.2, 21.9) | 69/435 (15.9) | 10.0 | 25.0 (19.3, 31.9) | 0.67 (0.46,0.98) | 0.362 |
| HFH > 12 months | 118/1188 (9.9) | 5.7 | 13.7 (11.2, 16.9) | 136/1129 (12.0) | 7.0 | 16.9 (14.1, 20.2) | 0.82 (0.64,1.04) |  |
| Never had a HFH | 126/1426 (8.8) | 5.3 | 13.7 (11.1, 16.8) | 138/1473 (9.4) | 5.6 | 14.5 (11.9, 17.6) | 0.92 (0.73,1.18) |  |
| **Baseline LVEF** | | | | | | | | |
| <median 31% | 162/1504  (10.8) | 6.4 | 15.5  (12.9, 18.5) | 206/1507 (13.7) | 8.3 | 20.6  (17.7, 23.9) | 0.77 (0.62,0.94) | 0.259 |
| ≥median 31% | 130/1548  (8.4) | 5.0 | 12.7  (10.4, 15.4) | 140/1542 (9.1) | 5.4 | 13.2  (11.0, 15.9) | 0.92 (0.72,1.17) |  |
| **Baseline eGFR** | | | | | | | | |
| 15-30 | 27/118  (22.9) | 15.1 | 30.2  (21.2, 42.0) | 23/125 (18.4) | 11.2 | 21.2  (14.2, 31.0) | 1.40 (0.80,2.44) | 0.073 |
| 31-60 | 93/921  (10.1) | 5.9 | 15.2  (12.0, 19.2) | 128/940 (13.6) | 8.3 | 18.8  (15.7, 22.4) | 0.70 (0.53,0.91) |  |
| >60 | 165/1954  (8.4) | 5.0 | 12.5  (10.4, 15.0) | 188/1921 (9.8) | 5.8 | 15.6  (13.3, 18.4) | 0.87 (0.70,1.07) |  |
| **NYHA Class at Baseline** | | | | | | | | |
| Class II | 200/2411 (8.3) | 4.9 | 12.3  (10.5, 14.4) | 229/2411 (9.5) | 5.7 | 14.2  (12.2, 16.5) | 0.87 (0.72,1.05) | 0.461 |
| Class III/IV | 92/642 (14.3) | 8.6 | 20.2  (16.0, 25.3) | 117/641 (18.3) | 11.2 | 26.4  (22.0, 31.6) | 0.76 (0.58,1.00) |  |
| **NT-pro BNP at Baseline (pg/mL)** | | | | | | | | |
| Q1 (≤827) | 32/753 (4.2) | 2.5 | 9.0  (5.7, 14.2) | 44/745  (5.9) | 3.4 | 9.7  (6.9, 13.5) | 0.73 (0.46,1.15) | 0.740 |
| Q2 (827 - 1375) | 57/752 (7.6) | 4.4 | 11.5  (8.4, 15.6) | 58/743  (7.8) | 4.5 | 11.3  (8.4, 15.2) | 0.98 (0.68,1.42) |  |
| Q3 (1375 - 2393) | 68/767 (8.9) | 5.2 | 13.2  (10.1, 17.2) | 74/730 (10.1) | 6.2 | 14.5  (11.3, 18.6) | 0.83 (0.60,1.16) |  |
| Q4 (>2393) | 130/727 (17.9) | 11.1 | 23.0  (19.3, 27.3) | 165/768 (21.5) | 13.9 | 32.7  (27.7, 38.2) | 0.80 (0.63,1.00) |  |

CI = confidence interval, eGFR = estimated glomerular filtration rate, GDMT = guideline-directed medical therapy, HFH = heart failure hospitalization, HR = hazard ratio, ICD = implantable cardioverter defibrillator, KM = Kaplan Meier estimate, NE = Not Estimable LVEF = left ventricular ejection fraction, PY = patient-year, SGLT2i = sodium-glucose cotransporter 2 inhibitor

**Table S2**. Mortality Subgroup Analysis: Time to All-Cause Death

|  | **Vericiguat (n=3,053)** | | | **Placebo (n=3,052)** | | | **Treatment Comparison** | |
| --- | --- | --- | --- | --- | --- | --- | --- | --- |
|  | n/m  (%) | Events/  100 PY | KM %  (95% CI) | n/m  (%) | Events/  100 PY | KM %  (95% CI) | HR  (95% CI) | Treatment by Subgroup P-Value |
| **Overall** | | | | | | | | |
|  | 377 /3053  ( 12.3) | 7.3 | 18.3  (16.3, 20.5) | 440 /3052 (14.4) | 8.6 | 20.6  (18.6, 22.8) | 0.84  (0.74, 0.97) |  |
| **Sex** | | | | | | | | |
| Male | 300/2326  (12.9) | 7.6 | 19.3  (17.0, 21.9) | 348/2339 (14.9) | 8.9 | 21.0  (18.8, 23.5) | 0.85 (0.73,0.99) | 0.870 |
| Female | 77/727  (10.6) | 6.3 | 14.8  (11.5, 19.0) | 92/713 (12.9) | 7.8 | 19.1  (15.1, 24.1) | 0.82 (0.61,1.12) |  |
| **Age Group (Years)** | | | | | | | | |
| < 65 | 107/1109  (9.6) | 5.7 | 15.2  (12.0, 19.3) | 133/1102 (12.1) | 7.2 | 17.7 (  14.6, 21.4) | 0.79 (0.61,1.01) | 0.512 |
| ≥ 65 | 270/1944  (13.9) | 8.2 | 20.0  (17.6, 22.7) | 307/1950 (15.7) | 9.4 | 22.2  (19.7, 25.0) | 0.87 (0.74,1.02) |  |
| **Race** | | | | | | | | |
| White | 248/1942  (12.8) | 7.3 | 17.6  (15.4, 20.1) | 286/1992 (14.4) | 8.3 | 20.3  (17.9, 22.9) | 0.88 (0.74,1.04) | 0.727 |
| Black | 33/251  (13.1) | 8.5 | 22.7  (14.8, 33.8) | 39/218 (17.9) | 11.5 | 28.0  (19.8, 38.5) | 0.73 (0.46,1.16) |  |
| Asian | 47/383  (12.3) | 7.7 | 16.0  (11.7, 21.6) | 50/363 (13.8) | 8.6 | 17.8  (13.3, 23.4) | 0.89 (0.60,1.33) |  |
| Other | 49/476  (10.3) | 6.4 | 22.0  (15.2, 31.3) | 65/479 (13.6) | 8.8 | 19.9  (15.1, 26.0) | 0.72 (0.50,1.05) |  |
| **Geographic Region** | | | | | | | | |
| Eastern Europe | 121/823  (14.7) | 8.4 | 20.3  (16.8, 24.4) | 149/877 (17.0) | 9.9 | 23.9  (20.3, 28.0) | 0.85 (0.67,1.08) | 0.598 |
| Western Europe | 64/586  (10.9) | 6.2 | 15.0  (11.4, 19.6) | 62/541 (11.5) | 6.4 | 15.1  (11.5, 19.6) | 0.95 (0.67,1.35) |  |
| North America | 41/324  (12.7) | 7.5 | 19.7  (14.1, 27.2) | 55/325 (16.9) | 10.2 | 23.5  (17.9, 30.5) | 0.74 (0.50,1.11) |  |
| Latin and South America | 97/888  (10.9) | 6.8 | 17.8  (13.8, 22.7) | 123/887 (13.9) | 8.8 | 21.7  (17.3, 27.0) | 0.75 (0.58,0.98) |  |
| Asia Pacific | 54/432  (12.5) | 7.8 | 18.1  (13.2, 24.5) | 51/422 (12.1) | 7.5 | 15.5  (11.7, 20.5) | 1.05 (0.72,1.54) |  |
| **Number of Baseline GDMT** | | | | | | | |  |
| 0–2 | 88/506  (17.4) | 9.9 | 22.3  (18.0, 27.5) | 101/501 (20.2) | 12.2 | 26.5  (21.7, 32.1) | 0.81 (0.61,1.08) | 0.950 |
| 3 | 149/1183  (12.6) | 7.3 | 18.1  (15.2, 21.5) | 174/1203 (14.5) | 8.4 | 20.0  (17.1, 23.3) | 0.86 (0.69,1.07) |  |
| 4 | 140/1364  (10.3) | 6.3 | 17.0  (13.8, 21.0) | 165/1348 (12.2) | 7.5 | 18.8  (15.8, 22.3) | 0.84 (0.67,1.05) |  |
| **Anemia** | | | | | | | | |
| Yes | 56/355  (15.8) | 9.3 | 25.7  (19.1, 33.9) | 84/398 (21.1) | 13.4 | 28.8  (22.9, 35.8) | 0.67 (0.48,0.95) | 0.151 |
| No | 321/2698  (11.9) | 7.1 | 17.3  (15.2, 19.6) | 356/2654 (13.4) | 8.0 | 19.5  (17.4, 21.8) | 0.88 (0.76,1.03) |  |
| **Diabetes** | | | | | | | | |
| Yes | 176/1274  (13.8) | 8.1 | 18.8  (16.1, 22.0) | 219/1311 (16.7) | 10.0 | 23.7  (20.5, 27.4) | 0.80 (0.66,0.98) | 0.463 |
| No | 201/1779  (11.3) | 6.7 | 17.9  (15.2, 21.0) | 221/1741 (12.7) | 7.6 | 18.2  (15.8, 21.0) | 0.89 (0.73,1.07) |  |
| **Atrial Fibrillation** | | | | | | | | |
| Yes | 155/1135  (13.7) | 7.9 | 18.6  (15.8, 21.9) | 195/1182 (16.5) | 9.7 | 22.3  (19.3, 25.6) | 0.81 (0.66,1.00) | 0.632 |
| No | 222/1918  (11.6) | 7.0 | 18.1  (15.5, 21.2) | 245/1870 (13.1) | 8.0 | 19.6  (16.9, 22.6) | 0.87 (0.73,1.04) |  |
| **Chronic Kidney Disease** | | | | | | | | |
| Yes | 85/520  (16.3) | 9.9 | 23.7  (18.7, 29.9) | 104/566 (18.4) | 11.0 | 26.0  (21.4, 31.4) | 0.91 (0.68,1.21) | 0.597 |
| No | 292/2533  (11.5) | 6.8 | 17.1  (15.0, 19.5) | 336/2486 (13.5) | 8.1 | 19.3  (17.1, 21.7) | 0.83 (0.71,0.97) |  |
| **Ischemic Heart Disease** | | | | | | | | |
| Yes | 250/1965  (12.7) | 7.4 | 18.8  (16.4, 21.6) | 284/1981 (14.3) | 8.5 | 20.1  (17.7, 22.7) | 0.86 (0.73,1.02) | 0.710 |
| No | 127/1088  (11.7) | 7.1 | 17.1  (13.9, 20.8) | 156/1071 (14.6) | 8.9 | 21.7  (18.3, 25.7) | 0.81 (0.64,1.03) |  |
| **Recency of HFH prior to randomization** | | | | | | | | |
| HFH 6–12 months | 62/424 (14.6) | 9.2 | 21.9 (16.6, 28.5) | 82/435 (18.9) | 11.9 | 27.8 (22.1, 34.6) | 0.78 (0.56,1.08) | 0.562 |
| HFH > 12 months | 144/1188 (12.1) | 7.0 | 17.6 (14.6, 21.1) | 170/1129 (15.1) | 8.8 | 20.7 (17.7, 24.1) | 0.80 (0.64,0.99) |  |
| Never had a HFH | 167/1426  (11.7) | 7.0 | 17.6  (14.8, 20.9) | 184/1473 (12.5) | 7.5 | 18.4  (15.5, 21.7) | 0.92 (0.75,1.13) |  |
| **Baseline LVEF** | | | | | | | | |
| <median 31% | 202/1504  (13.4) | 8.0 | 19.5  (16.6, 22.8) | 256/1507 (17.0) | 10.3 | 24.5  (21.4, 27.9) | 0.77 (0.64,0.93) | 0.155 |
| ≥median 31% | 175/1548  (11.3) | 6.7 | 17.1  (14.4, 20.1) | 184/1542 (11.9) | 7.1 | 16.8  (14.4, 19.7) | 0.94 (0.77,1.16) |  |
| **Baseline eGFR** | | | | | | | | |
| 15-30 | 36/118  (30.5) | 20.1 | 38.3  (28.5, 50.0) | 28/125 (22.4) | 13.6 | 24.3  (16.9, 34.1) | 1.53 (0.93,2.50) | 0.024 |
| 31-60 | 130/921  (14.1) | 8.3 | 21.6  (17.9, 26.0) | 173/940 (18.4) | 11.3 | 25.0  (21.4, 29.0) | 0.72 (0.57,0.90) |  |
| >60 | 203/1954  (10.4) | 6.2 | 15.4  (13.1, 18.1) | 231/1921 (12.0) | 7.1 | 18.3  (15.8, 21.1) | 0.87 (0.72,1.05) |  |
| **NYHA Class at Baseline** | | | | | | | | |
| Class II | 257/2411 (10.7) | 6.3 | 15.7  (13.7, 18.0) | 301/2411 (12.5) | 7.4 | 17.8  (15.7, 20.1) | 0.85 (0.72,1.00) | 0.954 |
| Class III/IV | 120/642 (18.7) | 11.2 | 26.9  (22.1, 32.5) | 139/641 (21.7) | 13.3 | 30.7  (25.9, 36.1) | 0.84 (0.66,1.07) |  |
| **NT-pro BNP at Baseline (pg/mL)** | | | | | | | | |
| Q1 (≤827) | 50/753 (6.6) | 3.9 | 12.9  (9.1, 18.1) | 58/745 (7.8) | 4.5 | 13.1  (9.8, 17.6) | 0.86 (0.59,1.26) | 0.640 |
| Q2 (827 - 1375) | 77/752 (10.2) | 5.9 | 16.3  (12.5, 21.2) | 78/743 (10.5) | 6.0 | 14.2  (11.1, 18.2) | 0.99 (0.72,1.36) |  |
| Q3 (1375 - 2393) | 85/767 (11.1) | 6.5 | 16.3  (12.9, 20.6) | 102/730 (14.0) | 8.5 | 19.6  (16.0, 24.0) | 0.76 (0.57,1.01) |  |
| Q4 (>2393) | 158/727 (21.7) | 13.5 | 28.0  (24.0, 32.6) | 197/768 (25.7) | 16.6 | 36.2  (31.4, 41.6) | 0.81 (0.66,1.00) |  |

CI = confidence interval, eGFR = estimated glomerular filtration rate, GDMT = guideline-directed medical therapy, HFH = heart failure hospitalization, HR = hazard ratio, ICD = implantable cardioverter defibrillator, KM = Kaplan Meier estimate, NE = Not Estimable, LVEF = left ventricular ejection fraction, PY = patient-year, SGLT2i = sodium-glucose cotransporter 2 inhibitor
