# Supplementary material for: Vericiguat and mortality in heart failure and reduced ejection fraction: the VICTOR trial
Source: Eur Heart J. 2025 Aug 30;47(6):683–97. doi: 10.1093/eurheartj/ehaf655 (PMC13089535; doi:10.1093/eurheartj/ehaf655)
Supplement: ehaf655_Supplementary_Data [file ehaf655_Supplementary_Data.zip › VICTOR_Study_Group.docx]

VICTOR Study Group

| **First Name** | **Surname** |
| --- | --- |
| Angela | Romero Zarante |
| Yenisca | Zambrano |
| Miguel | Hominal |
| Martin | Suarez |
| Ezequiel | Vottero |
| Marcia | Lopez |
| Oscar | Montaña |
| Gloria | Cendali |
| Marisa | Vico |
| Erica | Giozzi |
| Diego | Aizenberg |
| Santiago | Coussio |
| Diego | Arakaki |
| Miriam | Gelersztein |
| Scott | McKenzie |
| Kathryn | Stibilj |
| David | Colquhoun |
| Gemma | Macdonald |
| John | Atherton |
| Lydia | Lilwall |
| Carmine | De Pasquale |
| Simeoni | Thomas |
| Alicia | Chan |
| Denise | Healy |
| Brendan | McQuillan |
| Natalya | Beer |
| Dirk | von Lewinski |
| Viktoria | Rausch |
| Andreas | Schober |
| Maria-Christine | Leitgeb |
| Christopher | Adlbrecht |
| Cordula | Koehler |
| Johann | Auer |
| Manuela | Rossmaier |
| Moritz | Mirna |
| Kristen | Kopp |
| Jose | Saraiva |
| Carla Cristina | Vicente |
| Pedro | Schwartzmann |
| Gabriela | Ilana |
| Aguinaldo | Freitas |
| Sandra | Costa |
| Lidia | Moura |
| Fernanda | de Liz |
| Ricardo | Mourilhe Rocha |
| Roberta | Coelho |
| Fabiana | Marcondes-Braga |
| Talita | Antunes |
| Marcus | Simoes |
| Veronica | Silva |
| Joao | Souza |
| Grazielle | Freire |
| Fábio | Silveira |
| Tamyres | Galvão |
| Euler | Manenti |
| Carollainy | Silva |
| Ronald | Bourgeois |
| Karen | Boyd |
| Thao | Huynh |
| Caroline | Boudreault |
| Michael | Hartleib |
| Rebecca | Otis |
| James | Cha |
| Judy | Otis |
| Michael | Heffernan |
| Marie | Birch |
| A. Shekhar | Pandey |
| Samantha | Armstrong |
| Ashok | Mukherjee |
| Lubna | Zakria |
| Marie-Claude | Parent |
| Helene | Brown |
| Doug | Hayami |
| Deborah | Keller |
| Stephen Allan | Schaffer |
| Wendy | Janz |
| Yves | Pesant |
| Yanick | Sardin-Laframboise |
| Quentin | Kushnerik |
| Annie | Roy |
| Caroline | Boudreault |
| Gilbert | Gosselin |
| Nathalie | Leblanc |
| Yaariv | Khaykin |
| Debbie | Nemtean |
| Gordon | Moe |
| Carlos | Fernando |
| Ying | Sia |
| Amelie | Bujold |
| Jonathan | Howlett |
| Anusha | Sasikumar |
| Elizabeth | Swiggum |
| Noreen | Lounsbury |
| Serge | Lepage |
| Carl | Fortier |
| Subodh | Verma |
| Debbie | Chen |
| Fernando | Lanas |
| Pamela | Figueroa |
| Gabriel | Maluenda |
| Maria | Vergara |
| Juan | Prieto |
| Viviana | Noriega |
| Luis | Morales |
| Jessica | Zapata |
| Patricio | Yovaniniz |
| Marcela | Grandon |
| Franco | Appiani |
| Tatiana | del Rio |
| Sergio | Mellado |
| Claudia | Alvarez |
| German | Cruz |
| Sebastian | Mino |
| Juan Carlos | Palma Carvajal |
| Veronica | Olguin |
| Paz | Bourke |
| Maribel | Toledo Grell |
| Hugo | Marin |
| Sebastian | Rios |
| Manuel | Novajas |
| Macarena | Carcamo Medina |
| Jingmin | Zhou |
| Mingfeng | Lu |
| Jiyan | Chen |
| Yihan | Jiang |
| Junkui | Wang |
| Yuhong | Kang |
| Luping | Jiang |
| Yuxin | Zhong |
| Wei | Jin |
| Shanshan | Jin |
| Jianzeng | Dong |
| Jiajia | Wang |
| Jianqiang | Peng |
| Xiaomei | Peng |
| Aijie | Hou |
| Ruili | Duan |
| Weihong | Jiang |
| Xiaowei | Zhou |
| Xiang | Gu |
| Wenjuan | Dai |
| Yibin | Mei |
| Miaoxia | Liu |
| Jing | Yu |
| Jinxia | Chen |
| Zuyi | Yuan |
| Yuhong | Kang |
| Aidong | Shen |
| Yutao | Wang |
| Ruiping | Zhao |
| Peiyan | Ren |
| Zhuhua | Yao |
| Xiying | Zhang |
| Jingfeng | Wang |
| Huijun | Ouyang |
| Maohua | Chen |
| Yu | Lu |
| Peizhi | Miao |
| Zhenfei | Chen |
| Zhouqing | Huang |
| Xiaoli | Teng |
| Jiahong | Wang |
| Xuyao | Yan |
| Biao | Xu |
| Yue | Gao |
| Ping | Qiao |
| Mizozhu | Han |
| Mingya | Liu |
| Mingya | Liu |
| Shaorong | Wu |
| Ruihao | Li |
| Qian | Liang |
| Zhengzheng | Ni |
| Qilin | Ma |
| Ting | Li |
| Hongliang | Cong |
| Chuangting | Zhu |
| Guangping | Li |
| Lin | Liu |
| Weimin | Li |
| Junsheng | Jing |
| Qing | Zhang |
| Jia | Tang |
| Cuimei | Zhao |
| Liao | Ting |
| Xiaosheng | Hu |
| Yao | Fang |
| Yuehui | Yin |
| Xuejun | Liu |
| Jun | Jiang |
| Mengqing | Zhang |
| Yuanyuan | Luo |
| Siqi | Wang |
| Ping | Zhang |
| Peng | Zhang |
| Yanqing | Wu |
| Wenting | Wei |
| Xiaohua | Pang |
| Yanqiong | Xia |
| Lihong | Wang |
| Chenyu | Lv |
| Zhihua | Fang |
| Qiaochu | Du |
| Ping | Yang |
| Xuejiao | Liu |
| Wei | Yuan |
| Min | Wang |
| Kai | Huang |
| Danli | Xiang |
| Likun | Ma |
| Jinxiu | Liu |
| Claudia | Garcés Restrepo |
| Jose | Accini |
| Yineth | Conrado Ortega |
| Rodrigo | Botero |
| Aleydy | Caceres |
| Alberto | Cadena |
| Jessica | Martinez |
| Miguel | Moncada Corredor |
| Leisa | Florez |
| Camilo | Arana |
| Lizzette | Diaz Villegas |
| Dora | Molina de Salazar |
| Maria | Valencia |
| Juan | Carvajal |
| Paola | Delgado |
| Luis | Echeverria |
| Karen | Bueno Diaz |
| Juan | Gomez-Mesa |
| Laura | Trujillo |
| Eduardo | Echeverry |
| Adriana | Escruceria |
| Melissa | Reyes Dia |
| Adriana | Cervantes Hurtado |
| Ladislav | Busak |
| Blanka | Pokorna |
| Vojtech | Melenovsky |
| Anna | Kuprova |
| Martin | Hajsl |
| Jana | Brindova |
| Roman | Miklik |
| Veronika | Musilova |
| Libor | Nechvatal |
| Martin | Radvan |
| Renata | Machova |
| Jan | Belohlavek |
| Petra | Zavadilova |
| Zdenek | Monhart |
| Lenka | Bouzkova |
| Tomas | Drasnar |
| Matej | Fald |
| Nora | Bolvanska |
| Helena | Packova |
| Jan | Krejci |
| Jana | Roche |
| Jens | Hove |
| Lotte | Ring |
| Morten | Lindhardt |
| Therese | Bang-Hansen |
| Jens | Due Lomholdt |
| Kari | Niemann |
| Kristian | Madsen |
| Joan | Hummelshoej |
| Kenneth | Egstrup |
| Linda | Hindsgaul |
| Morten | Schou |
| Jette | Madsen |
| Søren | Nielsen |
| Emilie | Jakobsen |
| Henrik | Wiggers |
| Marianne | Leth |
| Lars | Kober |
| Elsebet | Eriksen |
| Niels | Eske Bruun |
| Winnie | Kragh |
| Jens | Bronnum-Schou |
| Pia | Hadsund |
| Henrik | Vadmann |
| Annette | Nielsen |
| Michel | Galinier |
| Nathalie | Rosolin |
| Jean-Michel | Tartiere |
| Pauline | Armangau |
| Emile | Ferrari |
| Nassima | Belkacem |
| Cadi | Aludaat |
| Line | Balaya-Gouraya |
| Emmanuelle | Berthelot |
| Agnes | Pezzi |
| Nicolas | Girerd |
| Emilie | Tachot |
| Teodora | Dutoiu |
| Anne-Sophie | Derien |
| Emmanuel | Boiffard |
| Philippe | Prunier |
| Francois | Picard |
| Manel | Moumni |
| Richard | Isnard |
| Ghoulem | Tabdjoun |
| Nathan | Mewton |
| Bianca | Barry |
| Arne | Geissler |
| Jeanette | Schumacher |
| Peter | Schwimmbeck |
| Bianca | Krug-Hoeren |
| Dimitrios | Dimitroulis |
| Judith | Simons |
| Jan-Malte | Sinning |
| Julia | Müller |
| Martin | Duersch |
| Daniela | Leyh |
| Stefan | Stork |
| Elisa | Wied |
| Ulrich | Overhoff |
| Mariana | Rupprecht |
| Hannes | Lindemann |
| Ines | von Hertell |
| Werner | Rieker |
| Jelena | Anacker |
| Henning | Ebelt |
| Ute | Goebel |
| Alexander | Jabs |
| Stephanie | Kirchenwitz |
| Christian | Mahnkopf |
| Mario | Hahn |
| Martin | Bergmann |
| Cecile | Bosredon |
| Mirko | Brudzinski |
| Katja | Steinbruegger |
| Axel | Linke |
| Kerstin | Eck |
| Daniel | Beug |
| Maria | Gonzalo de Juan |
| Kerstin | Schadow |
| Nicole | Schaefer |
| Norbert | Frey |
| Benjamin | Schafer |
| Johann | Bauersachs |
| Alexandros | Markakis |
| Katrin | Eberhard |
| Maria | Karmpalioti |
| Dimitrios | Tziakas |
| Vaggelitsa-Eirini | Kesoglou |
| Konstantinos | Tsioufis |
| Ilias | Gkartzonikas |
| Sotirios | Patsilinakos |
| Pinelopi | Karagogou |
| Aikaterini | Naka |
| Petros | Nikolopoulos |
| Ioannis | Skoularigkis |
| Foteini | Ereliadou |
| Gerasimos | Filipatos |
| Paraskevi | Lathridou |
| Vlasios | Ninios |
| Evangelia | Androutsou |
| Haralampos | Karvounis |
| Vasiliki | Moutafi |
| Alexandros | Briasoulis |
| Christin | Mandila |
| Elias | Tsougos |
| Lissette | Garcia de Krumbach |
| Apostolos | Karavidas |
| Mayra | Aguilar Schinini |
| Juan | Arango Benecke |
| Andrea Lucia | Ramírez Ávila |
| Pablo | Montenegro Valdovinos |
| Xue | Ting Wang |
| Marco | Rodas Diaz |
| Venus | San Lui Ho |
| Pui-Wai Alex | Lee |
| Eva | Tam |
| Siu Han Jojo | Hai |
| Kar Wai | Lee |
| Katherine Yue Yan | Fan |
| Zsolt | Nagy |
| Ngai-Yin | Chan |
| Karolyne | Rozsa Toth |
| Sandor | Kancz |
| Krisztina | Toth |
| Robert | Kirschner |
| Laura | Kósa-Hóbor |
| Ferenc | Lakatos |
| Barna | Bartha |
| Bela | Merkely |
| Gabriella | Bencze |
| Andras | Papp |
| Gabor | Kiss |
| Zsolt | Zilahi |
| Szilvia | Kovacs |
| Istvan | Kovacs |
| Szilvia | Levang |
| Tunde | Toth |
| Monika | Nagy |
| Tamas | Habon |
| Réka | Kónyi |
| Janos | Takacs |
| Nora | Hadi |
| Attila | Konyi |
| Katalin | Spandli |
| Bela | Benczur |
| Maria | Szabone Nagy |
| Botond | Literati-Nagy |
| Arpad | Kormanyos |
| Andras | Nagy |
| Gyongyver | Ibolya Mihaly |
| Robert | Sepp |
| Anita | Kalapos |
| Ebrahim | Noori |
| Jan | Mayene Abalos |
| Attila | Thury |
| Joanne | Maher |
| Ross | Murphy |
| Neda | Nabwani |
| Kenneth | McDonald |
| Hadas | Even Nir |
| Shaul | Atar |
| Galit | Paor |
| Tuvia | Ben Gal |
| Meital | Biener |
| Oren | Caspi |
| Shiree | Yedid Am |
| Sorel | Goland |
| Jenny | Elber |
| Tal | Hasin |
| Ira | Lapidus |
| Jameel | Mohsen |
| Dema | Barhum |
| Gil | Moravsky |
| Sapir | Swisa |
| Donna | Zwas |
| Nava | Eizenberg |
| Alexander | Goldman |
| Marilisa | Ambrosio |
| Alexander | Fardman |
| Noemi | Punzo |
| Irene | Mattavelli |
| Alessandro | Fucili |
| Luca | Assoni |
| Piergiuseppe | Agostoni |
| Valentina | Morsella |
| Savina | Nodari |
| Marta | Maffi |
| Maurizio | Volterrani |
| Ilaria | Gallelli |
| Laura | Scelsi |
| Michela | Algeri |
| Claudio | Borghi |
| Roberta | Famiani |
| Alessandro | Maloberti |
| Federica | Ramani |
| Stefano | Carugo |
| Francesca | Vannuccini |
| Gianfranco | Sinagra |
| Caterina | Scapicchi |
| Alberto | Palazzuoli |
| Giuditta | Cuccuru |
| Giuseppe | Ambrosio |
| Francesca | D'Alessandro |
| Gaetano | De Ferrari |
| Manhaiyun | Suhaimi |
| Roberto | Badagliacca |
| Juriah | Sulehan |
| Imran | Zainal Abidin |
| Fatma Diyana | Mohd Bukhari |
| Tiong Kiam | Ong |
| Nur | Alia |
| Khairani | Siti |
| Zainal | Abidin |
| Neoh Poh | Tiew |
| Shariff | Raja |
| Ezman | Faridz Shariff |
| Zalida | Mamat |
| Abd | Syukur Abdullah |
| Mabelle | Wong |
| W | Isa |
| W | Yus Haniff |
| Nurul | Suhaili Mamat |
| Houng Bang | Liew |
| Haydea | Hirata Avila |
| Ahmad | Wazi Ramli |
| Ma. | Aguilar Sanchez |
| Gustavo | Mendez Machado |
| Hannah | Guzman Solorzano |
| José | Arenas León |
| Michelle | Meza Hernandez |
| Maria | Arias Mendoza |
| Rosa | Flores Pruneda |
| Enrique | Lopez Rosas |
| Carlos | Castro Zarate |
| Alberto | Bazzoni Ruiz |
| Ana Rosa | Ramos Ruiz |
| Jesus | Perez Rios |
| Lucia | de la Vega |
| Armando | Segura Gonzalez |
| Wendy | Mendez |
| William | Gandoy Vazquez |
| Luz Belen | Garcia Gonzalez |
| Hilda | Peralta Rosado |
| Maria | Maytorena |
| Alberto | Varela Varela |
| Elena | Ruiz Rubio |
| Paul | Vazquez |
| Nazareth | Goiz |
| Jaime | Chavez Michel |
| Claudia | Lopez |
| Pedro | Fajardo Campos |
| Sergio | del Muro Munoz |
| Jesus | Illescas Diaz |
| America | Medina Reyes |
| Ricardo | Garcia |
| Araceli | Cuevas |
| Hector | Salazar Lopez |
| Ana | García Briones |
| Norberto | Matadamas |
| Edgar | Barcenas |
| Castelán | Vargas |
| Marcos | Astorga Rivas |
| Edmundo | Bayram Llamas |
| Felipe | Olguin Ortiz |
| Gerardo | Payro |
| Geraldine | González |
| Eduardo | Zambrano Ruiz |
| Arianna | Rodriguez Cal Y Mayor |
| Alejandro | Ricalde |
| Stephanie | Rose |
| Marcelo | Ramirez Mendoza |
| Jo-Anne | Kovacs |
| Richard | Troughton |
| Deborah | Scott |
| Katherine | Ferrier |
| Sharon | Jaques |
| James Pemberton | Pemberton |
| Cathy | Hulbert |
| Vijay | Dhakshinamurthy |
| Jamie | Duckworth |
| Nezar | Amir |
| Danica | Kistanna |
| Mansi | Turaga |
| Sonia | Romero |
| Jocelyne | Benatar |
| L. Jeanneth | Rodriguez |
| Aldo Edwin | Rodriguez Escudero |
| Jill | Espejo |
| Felix | Medina Palomino |
| Carola | Ariza Villanueva |
| Libia | Lu Galarreta |
| Milagros | Matta |
| Jose | Cabrera |
| J. | Zena |
| Norma | del Rosario |
| Armando | Godoy Palomino |
| Maria | Garcia |
| Roger | Correa |
| Leonila | Castillo |
| Percy | Berrospi |
| Maria | Gil Rescavarren |
| Luisa | Cardoza Anton |
| Bozena | Chromczak |
| Luis | Camacho Cosavalente |
| Paulina | Idzikowska |
| Zbigniew | Pijanowski |
| Joanna | Wilczynska |
| Grzegorz | Skoczylas |
| Monika | Tunkiewicz |
| Lidia | Pawlowicz |
| Alina | Cieszynska |
| Joanna | Szachniewicz |
| Wojciech | Zajdel |
| Krzysztof | Cymerman |
| Agnieszka | Polak |
| Wojciech | Zajdel |
| Marta | Augustyn |
| Aleksander | Zurakowski |
| Anna | Ryznar-Sicinska |
| Robert | Witek |
| Natalia | Markiewicz |
| Stanislaw | Mazur |
| Anna | Beme |
| Tomasz | Lugowski |
| Pawel | Walasek |
| Beata | Miklaszewicz |
| Urszula | Matys |
| Jacek | Gniot |
| Monika | Rolirad |
| Wanda | Sudnik |
| Dorota | Bartoszewska |
| Grzegorz | Skonieczny |
| Airleen | Camacho-Molina |
| Janusz | Prokopczuk |
| Brenda | Molina Elicier |
| Ismael | Toro-Grajales |
| Jose | Vazquez-Tanus |
| Noemi | Torres-Rivas |
| Evelyn | Matta Fontanet |
| Loriany | Alcocer Rodriguez |
| Yolanda | Figueroa-Torres |
| Andrei | Zhernokleev |
| Luis | Rodriguez-Ospina |
| Larisa | Konshina |
| Alexander | Vishnevskiy |
| Marina | Teterina |
| Yury | Shvarts |
| Elena | Volkova |
| Imad | Meray |
| Alla | Ledyaeva |
| lga | Bolshakova |
| Oxana | Evtushenko |
| Yury | Lopatin |
| Natalia | Peregudova |
| Alexander | Kastanayan |
| Sergey | Solnyshkov |
| Sergey | Yakushin |
| Irina | Garina |
| Elena | Shutemova |
| Larisa | Khaisheva |
| Irina | Kraeva |
| Natalya | Koziolova |
| Natalia | Kizhvatova |
| Dmitry | Kositsyn |
| Elena | Yakovleva |
| Elena | Kosmacheva |
| Dmitry | Dronov |
| Yury | Lukyanov |
| Olga | Sorokina |
| Dmitry | Dronov |
| Anna | Samodurova |
| Elena | Isaeva |
| Maria | Trukshina |
| Evgeniy | Kovalchuk |
| Nikloai | Ustiuzhanin |
| Maria | Sitnikova |
| Eveline | Febriana |
| Mikhail | Zykov |
| Xin Tian | Chua |
| Kheng Leng David | Sim |
| Kah Min | Ang |
| Seet Yoong | Loh |
| Siti Norashikin Binte | Fuad |
| Weiqin | Lin |
| Marelize | van der Mescht |
| Weiliang | Huang |
| Michelle | Pretorius |
| Lesley | Burgess |
| Louwra | Greyling |
| Muhammad | Ameen Fulat |
| Noloyiso | Mtana |
| Ellen | Makotoko |
| Deborah | Katisi |
| Mpiko | Ntsekhe |
| Nozibusiso | Mosia |
| Theema | Nunkoo |
| Vaman | Naidoo |
| Rosie | Mngqibisa |
| Sonnika | van Vuuren |
| Eric | Klug |
| Unarine | Madavha |
| Zarinah | Mohamed |
| Engela | Nortje |
| Paul | Rheeder |
| Tracy | Jacovides |
| Susan | Arnold |
| Chano | Stuurman |
| Andrew | Jacovides |
| Catharina | Faul |
| Kathleen | Coetzee |
| Zakariyyaa | Mohamed |
| Douwe | De Jong |
| Seul Gi | Park |
| Riaz | Dawood |
| WonMi | Lee |
| Myeong-Chan | Cho |
| Hwayoung | Jeong |
| Seok-Min | Kang |
| Ryeong Gyeong | Lee |
| Jung-Hyun | Choi |
| Seon Mi | Shin |
| Jin-Ok | Jeong |
| Eun | Kim |
| Byung-Su | Yoo |
| Boram | Park |
| Sang-Ho | Jo |
| Gaeul | Chae |
| Wook-Jin | Chung |
| MiJu | You |
| Hyun-Jai | Cho |
| Diego | Rodriguez Penas |
| Kye Hun | Kim |
| Catalina | Carrasco Ossorio |
| Jose Ramon | Gonzalez-Juanatey |
| Anna | Mollar Fernandez |
| Antonio | Reyes Dominguez |
| Ruben | Martinez Jaen |
| Julio | Nunez Villota |
| Judith | Viaplana |
| Ignacio | Ferreira |
| Isabel | Sicilia Bravo |
| Josep | Comin-Colet |
| Paz | Gonzalez Portilla |
| Josebe | Goirigolzarri Artaza |
| Cristina | Soler |
| Jesus | Alvarez Garcia |
| Paloma | Gastelurrutia |
| Nuria | Farre Lopez |
| Guiomar | Mediavilla Garcia |
| Antoni | Bayes Genis |
| Zulaica | Grille Cancela |
| Esteban | Lopez de Sa Areses |
| Kristina | From |
| Maria | Generosa Crespo Leiro |
| Pernilla | Haglund |
| Niklas | Bergh |
| Malin | Broberg |
| Barna | Szabo-Soderberg |
| Kristina | Eriksson |
| Carl-Johan | Lindholm |
| Carina | Andersson |
| Niclas | Svedberg |
| Isabel | Tronstad |
| Ingemar | Lonnberg |
| Håkan | Södergren |
| Emil | Najjar |
| Birgitta | Rangman |
| Marcus | Lind |
| Chiao-Wen | Chang |
| Ake | Olsson |
| Vinny | Chou |
| Chern-En | Chiang |
| Huey-Pyng | Su |
| Ming-En | Liu |
| Ping-Ping | Chou |
| I-Chang | Hsieh |
| Hui-Ling | Huang |
| Jin-Long | Huang |
| Hsiao-Ting | Lee |
| Tsung-Hsien | Lin |
| Akin | Aydin |
| Chien-Hsun | Hsia |
| Burcu | Kaygusuz |
| Yuksel | Cavusoglu |
| Selin | Yildirim |
| Bahar | Tak |
| Özge | Durhan |
| Sanem | Nalbantgil |
| Emel | Sahin |
| Ahmet | Celik |
| Ebru | Sahin |
| Hakan | Altay |
| Fatma | Nur Ertunc |
| Ersel | Onrat |
| Gizem | Celik Eminoglu |
| Cihan | Altin |
| Esmanur | Mizrak |
| Tayfun | Sahin |
| Bulut | Akbulut |
| Ergun | Baris Kaya |
| Ezgi | Aslan |
| Hasan | Barman |
| Berna | Akin |
| Vineet | Venugopal |
| Iulia | Volchyk |
| Thomas | Duythuc To |
| Taras | Chendey |
| Oleksandr | Kulbachuk |
| Mishka | Pavelko |
| Roman | Stets |
| Olena | Beregova |
| Mykola | Rishko |
| Oksana | Pysanko |
| Andriy | Yagensky |
| Yaroslav | Lutai |
| Leonid | Rudenko |
| Ganna | Rudya |
| Dmytro | Reshotko |
| Andriy | Yurkiv |
| Oleksandr | Parkhomenko |
| Oksana | Gliebova |
| Larysa | Mishchenko |
| Diana | Schyhypak |
| Olena | Levchenko |
| Halyna | Havryliuk |
| Oleksandr | Prokhorov |
| Oksana | Bilonko |
| Oleksandra | Donets |
| Alla | Dzyba |
| Halyna | Myshanych |
| Olha | Radchenko |
| Sergii | Shevchuk |
| Andrii | Danyliuk |
| Ivan | Vyshnyvetskyy |
| Tetiana | Danko |
| Vira | Tseluyko |
| Taras | Petrovskyy |
| Andrii | Klantsa |
| Volodymyra | Sovtus |
| Anna | Kulyk |
| Tetiana | Shandra |
| Roman | Petrovskyy |
| Elena | Romanova |
| Igor | Vakaliuk |
| Denys | Babichev |
| Svitlana | Zhurba |
| Ganna | Krotenko |
| Oleg | Sychov |
| Nataliia | Kumpan |
| Iurii | Rudyk |
| Svitlana | Trubina |
| Ozlem | Yildirimturk |
| Sandra | Elphick |
| Ahmet | Genc |
| Alexandra | Florou |
| Ahmet | Fuat |
| Rita | Adrego |
| Craig | Barr |
| Sinead | Lyons |
| Theresa | McDonagh |
| Sarah | Broadhead |
| Lisa | Anderson |
| Joanna | Brown |
| Justin | Cooke |
| Michelle | Kinnin |
| Christopher | Cassidy |
| Francis | Galera |
| Andrew | Moriarty |
| Abeesh | Panicker |
| Ify | Mordi |
| Lisa | Mellish |
| Manish | Saxena |
| Susan | Crawford |
| Prithwish | Banerjee |
| Rosemary | Harper |
| Matthew | Dewhurst |
| Deborah | Jones |
| Madhusudhan | Varma |
| Ben | Jones |
| Bassem | Ibrahim |
| Rebecca | Cutts |
| Aaron | Wong |
| Angela | Doughty |
| Rumi | Jaumdally |
| Susan | Regan |
| Patrick | Moore |
| Lani | Holman |
| David | Wilson |
| Cynthia | Larew |
| Patrick | Donnelly |
| Haydee | Gutierrez |
| Mohsin | Alhaddad |
| Phillippa | McCartney Morales |
| Linda | Cadaret |
| Jorge | Allina |
| Mehrdad | Ariani |
| Sajad | Hamal |
| Rasha | Youssef |
| Maite | Ramirez |
| David | Bernard |
| Madeline | Peek |
| Matthew | Budoff |
| Kelli | Clopp |
| Jose | Cardona |
| Jaime | Wyatt |
| James | Feldman |
| Stephanie | Lambert |
| Ferris | George |
| Beatriz | Penafiel |
| Eve | Gillespie |
| Marie | Reno |
| Obinna | Isiguzo |
| Linda | Schwarz |
| Luis | Martinez |
| Rose | Hroncich |
| Usman | Qayyum |
| Donna | Winterrowd |
| Renee | Sangrigoli |
| Sabrina | Forbus |
| Marcus | Williams |
| Kevin | Thornton |
| Ravi | Bhagwat |
| Crystal | Dubois |
| Brian | Foley |
| Heather | Maschenik |
| Ashish | Gangasani |
| Moravia | Vasallo |
| Naseem | Jaffrani |
| Anne | Dirks |
| Felix | Sogade |
| Lauren | Stamschror |
| Javier | Vasallo |
| Leigha | Hughes |
| Gregory | Ewald |
| Mohamed | Khalil |
| Majdi | Ashchi |
| Nancy | Fry |
| Sandeep | Khosla |
| Heather | Blubaugh |
| Hahn | Bui |
| Smelda | Ferrin |
| John | McGinty |
| Rafael | Muro |
| Jesse | Doran |
| Abeer | Abu-Saif |
| Dennis | Spiller |
| Jennifer | Bajornas |
| Hamid | Taheri |
| Marisol | Ramos |
| Imad | Hariri |
| Alicia | Phillips |
| Ataul | Qureshi |
| Jill | Schulte |
| Narendra | Singh |
| Vicky | Parfait |
| Andrea | Phillips |
| Malcolm | Lust |
| Juliane | Vierecke |
| Margie | McKercher |
| Peter | Fail |
| Jacqueline | Killian |
| Robert | Long |
| Victoria | Smith |
| Ramon | Lloret |
| Rema | Iskandarani |
| Nabil | Dib |
| Breanna | Culver |
| Siby | Ayalloore |
| Ali | Muhammad |
| Katherine | Michelis |
| Stephanie | Alford |
| Fahed | Bitar |
| Amy | King |
| Lokesh | Chandra |
| Richard | Jackson |
| Vijay | Rao |
| Hailey | Jenkins |
| David | Hotchkiss |
| Yulia | Abidov |
| Richard | Jackson |
| Ariel | Quebedeaux |
| Matthew | Janik |
| Sharyce | Hayes-Jones |
| Marcel | Zughaib |
| Teresa | Osmulski |
| Wade | May |
| Natalie | Fiallo |
| Dilip | Viswanath |
| Emily | Gilder |
| Alexander | Adler |
| Amanda | Reynolds |
| Joshua | Larned |
| Ashley | Williams |
| Joseph | Surber |
| April | Howarter |
| Mark | Napoli |
| Robert | Edmond |
| Wayne | Old |
| Alison | Solomon |
| Christopher | Sparrow |
| Mihret | Assefa |
| Divya | Gupta |
| Nicole | Scott |
| Glenn | Hamroff |
| Sirisha | Tirumanisetti |
| Chrisette | Dharma |
| Shelli | Roberts |
| William | Herzog |
| Lynett | Zachary |
| Iyad | Isseh |
| Loren | Moore |
| Christopher | Daniels |
| Kimberly | Armstrong |
| Lars | Runquist |
| Svetlana | Strugatsky |
| Ghiyath | Tabbal |
| Nehal | Vekariya |
| Andre | Artis |
| Ramona | Stevens |
| Freny | Mody |
| Ibraheem | AlAjlouni |
| Pankaj | Arora |
| Peter | Osmond |
| Mahfouz | El Shahawy |
| Yasmire | Evans |
| Ghiath | Mikdadi |
| Nadine | Kallas |
| Joseph | Izzo |
| Iluyomade | Adeusi |
| Anu | Lala-Trindade |
| Lisa | English |
| Brian | Bostick |
| Anastasiia | Piskun |
| Alexandra | Cornell |
| Constance | Hamlin |
| Katie | Lamont |
| Martina | Zilli |
| Christi | Kent |
| Jaco | De Klerk |
| Christina | Salerno |
| Lydia | Christopher |
| Marisa | Vico |
| Scott | McKenzie |
| Dirk | von Lewinski |
| Fabiana | Marcondes |
| Shelley | Zieroth |
| Anique | Ducharme |
| Fernando | Lanas |
| Junbo | Ge |
| Juan Esteban | Gómez |
| Vojtech | Melenovsky |
| Henrik | Wiggers |
| Michel | Galinier |
| Stefan | Stoerk |
| Gerasimos | Filippatos |
| Pablo | Montenegro |
| Alex | Lee |
| Sandor | Kancz |
| Ken | McDonald |
| Gil | Moravsky |
| Marco | Metra |
| Imran | Abidin |
| Richard | Troughton |
| Felix | Medina |
| Jose | Vazquez-Tanus |
| Yury | Lopatin |
| David | Sim |
| Karen | Sliwa |
| Myeong-Chan | Cho |
| Marisa | Leiro |
| Emil | Najjar |
| Chern-En | Chiang |
| Yüksel | Cavusoglu |
| Oleksandr | Parkhomenko |
| Craig | Barr |
| Palak | Shah |
| Matthew | Budoff |
| Barry | Greenberg |
| Tom D. | Cook |
| Aldo | Maggioni |
| Karl | Swedberg |
| Jane E. | Wilcox |
| Maria Rosa | Costanzo |
| Haley | Hedlin |
| W. Schuyler | Jones |
| Karen | Alexander |
| Sana | Al-Khatib |
| Jorge | Gutierrez |
| Robert | Harrison |
| Mark | Kittipibul |
| Bradley | Kolls |
| David | Kong |
| Joseph | Lerman |
| Robert | Matthews |
| Robert | McGarrah |
| Rajendra | Mehta |
| Thomas | Povsic |
| Jennifer | Rymer |
| Cina | Sasannejad |
